# Safety and efficacy associated with single-fraction high-dose-rate brachytherapy in localized prostate cancer: a systematic review and meta-analysis

**DOI:** 10.1007/s00066-023-02063-z

**Published:** 2023-04-24

**Authors:** Hong Zeng, Jindong Dai, Dehong Cao, Minghao Wang, Jinge Zhao, Yuhao Zeng, Nanwei Xu, Yandong Xie, Haolin Liu, Hao Zeng, Guangxi Sun, Pengfei Shen

**Affiliations:** Department of Urology, Institute of Urology, West China Hospital, Sichuan University, Chengdu, China

**Keywords:** Prostatic neoplasms, Radiation dose hypofractionation, Monotherapy, Adverse effects, Clinical benefit

## Abstract

**Objective:**

Although single-fraction high-dose-rate brachytherapy (SFHDR) for localized prostate cancer has been tried in clinical trials, relevant medical evidence is currently lacking. It is necessary to systematically analyze the safety and efficacy of SFHDR.

**Methods:**

Comprehensive and systematic searches for eligible studies were performed in PubMed, Embase, and the Cochrane Library databases. The primary endpoints included safety and efficacy, represented by toxic effects and biochemical recurrence-free survival (bRFS), respectively. The proportion rates were used as the effect measure for each study and were presented with corresponding 95% confidence intervals (CI) and related 95% prediction interval (PI). Restricted maximum-likelihood estimator (REML) and the Hartung–Knapp method were used in the meta-analysis.

**Results:**

Twenty-five studies met the inclusion criteria for quantitative analysis, including 1440 patients. The median age of patients was 66.9 years old (62–73 years old) and the median follow-up was 47.5 months (12–75 months). The estimates of cumulative occurrence for severe gastrointestinal (GI) and genitourinary (GU) toxic effects were 0.1% (95% CI 0–0.2%) and 0.4% (95% CI 0–1.2%), and for grade 2 toxic effects were 1.6% (95% CI 0.1–4.7%) and 17.1% (95% CI 5.4–33.5%), respectively. The estimate of 3‑year bRFS was 87.5% (95% CI 84.4–90.3%) and 71.0% (95% CI 63.0–78.3%) for 5‑year bRFS. The pooled bRFS rates for low-risk patients were 99.0% (95% CI 85.2–100.0%) at 3 years and 80.9% (95% CI 75.4–85.9%) at 5 years, and the risk group was found to be statistically correlated with bRFS (3-year bRFS, *P* < 0.01; 5‑year bRFS, *P* = 0.04).

**Conclusion:**

SFHDR is associated with favorable tolerability and suboptimal clinical benefit in patients with localized prostate cancer. Ongoing and planned high-quality prospective studies are necessary to verify its safety and efficacy.

**Supplementary Information:**

The online version of this article (10.1007/s00066-023-02063-z) contains supplementary material, which is available to authorized users.

## Main novel aspects

- Single-fraction high-dose-rate brachytherapy (SFHDR) for localized prostate cancer shows favorable safety given the low incidence of toxic events.
- Low-risk patients seem to be suited for SFHDR.

## Introduction

Treatment options for definitive radiotherapy of prostate cancer can be performed with commonly used external-beam radiation therapy (EBRT) and low-dose-rate brachytherapy (LDRB). As a safe and effective method of dose escalation, high-dose-rate brachytherapy (HDRB) is also emerging, allowing for highly conformal coverage of the prostate while minimizing the dose to surrounding organs at risk (OAR) [1]. According to the German S3 guidelines, HDRB is recommended in combination with EBRT for the treatment of eligible patients with localized prostate cancer. However, mature results from clinical trials have demonstrated that multi-fraction HDRB as monotherapy in localized prostate cancer can achieve comparable disease control to LDRB and EBRT [2–5].

Over the past practice, some shortcomings of multi-fraction HDRB have been noticed. Frequent hospitalizations, multiple implants, and bed rest may be required during the treatment process. Due to resource consumption and logistic challenges, the appeal of multi-fraction HDRB tends to be detracted, especially compared to the permanent seed implant of LDRB [6, 7]. In addition, regarding radiobiological considerations, the low α/β ratio and high sensitivity to hypo-fractionated radiotherapy in prostate cancer have led to increased interest in single-fraction HDRB (SFHDR) [8–10]. Such an approach may make HDRB favorable to LDRB in that SFHDR reduces the need for multiple implants and is more attractive in terms of practicality, convenience, toxicity [11, 12].

Currently, however, SFHDR is not generally applied to localized prostate cancer patients and there are still controversies regarding the optimal dose regimen and selection of disease risk group. Assuming that SFHDR is a potential curative alternative for localized prostate cancer, we aim to better understand the safety and efficacy of the therapy through this latest systematic review and meta-analysis.

## Materials and methods

### Search strategy and study selection

The Preferred Reporting Items for Systematic Reviews and Meta-Analyses (PRISMA) statement was applied to this systematic review and meta-analysis [13]. We registered it in the International Prospective Register of Systematic Reviews (PROSPERO) of the Centre for Review and Dissemination (CRD42022319328). The participants, intervention(s), control, outcomes, study design (PICOS) approach [14, 15] was used to set the inclusion criteria for the literature (eTable 1 in the Supplement).

The inclusion criteria for the studies were as follows: 1) patients with localized prostate cancer who were primarily treated with HDRB radiotherapy; 2) prospective or retrospective randomized or non-randomized studies with a single group or multiple groups; 3) all patients in the treatment group received a single dose of HDRB (defined as more than 15 Gy per fraction), with or without androgen deprivation therapy (ADT); 4) at least one major endpoint measure was reported, including gastrointestinal (GI) toxicity, genitourinary (GU) toxicity, 3‑year bRFS, and 5‑year bRFS. Exclusion criteria included 1) non-human experimental studies, 2) patients who received adjuvant radiotherapy or had metastatic prostate cancer or developed disease relapse, 3) non-English articles, and 4) sample size fewer than 15.

Two investigators (M.H.W. and J.D.D.) conducted independent, comprehensive, and systematic searches in PubMed, Embase, and the Cochrane Library databases. The main retrieval strategies included the following different terms: “prostate” and “cancer or neoplasms” and “single or one” and “dose or fraction” and “high dose rate or HDR” and “brachytherapy.” In addition, potentially relevant literature was tracked through articles or reviews to supplement appropriate studies for the meta-analysis. Detailed search strategies are available at PROSPERO according to the CRD number. The searches were reconducted to complement the study before the final analysis and the final search was conducted in October 2022.

### Data extraction

Data extraction was performed based on a pre-designed standardized form for the included studies. This process was independently conducted by two reviewers (N.W.X. and J.G.Z.). A third investigator (G.X.S.) was involved in resolving any differences between the two reviewers. Extracted variables included study design, country, time of the study, sample size, dose per fraction, HDRB image guiding method, dose constraints, median follow-up time, and patient characteristics before treatment (such as median age, prostate volume, PSA level, ADT usage, clinical tumor stage, Gleason score, and risk group), toxic events rate (GU and GI toxicity), 3‑year bRFS, and 5‑year bRFS. In the absence of specific bRFS data for articles, Plot Digitizer version 2.6.8 (SourceForge, Boston, Massachusetts, the United States) was used to extract values from Kaplan–Meier curves.

In addition, two researchers (Y.H.Z. and Y.D.X.) recorded the risk of bias assessment results for each study. The revised Cochrane risk-of-bias tool for randomized trials (RoB 2) was used to evaluate randomized trial studies. This consists of five domains, including randomization process, intended interventions, missing outcome data, measurement of the outcome, and selection of the reported result [16]. For non-randomized experimental studies, methodological quality was assessed using the methodological index for non-randomized studies (MINORS) [17].

### Outcomes

The primary outcome measure was safety, represented by GU and GI toxic event rates, and the secondary outcome measure was efficacy, represented by bRFS rates. Toxic event rates involve severe and grade 2 toxicity, where severe toxicity was defined as an event greater than or equal to grade 3, primarily based on the Radiation Therapy Oncology Group (RTOG) or the Common Terms for Adverse Events (CTCAE). Any toxic event requiring hospitalization or surgical intervention, or directly reporting “severe” toxicity, was considered as a grade ≥ 3 toxicity. Studies reporting toxicity events that were not distinguished as GU or GI toxicity were excluded. Nevertheless, if the toxic event was zero, both GU and GI toxic effects were included in the analysis at zero.

Efficacy was evaluated by 3‑year bRFS and 5‑year bRFS. Although different articles described biochemical failure in different ways, such as biochemical no evidence of disease (bNED), biochemical disease-free survival (bDFS), biochemical control rate (BCR), biochemical progression-free survival (bPFS), and biochemical failure-free survival (BFFS), they were all based on the Phoenix definition (a rise by 2 ng/mL or higher above the PSA nadir) to define biochemical failure (BF) [18]. Therefore, these outcomes were considered equivalent for bRFS.

### Statistical analysis

The proportion rates of patients who experienced particular events were used as the effect measure for each study and were presented in a forest plot with corresponding 95% confidence intervals (CI) and related 95% prediction interval (PI). For any study with an event rate of 0 or 1, a continuity correction of 0.5 was applied. Restricted maximum-likelihood estimator (REML) and the Hartung–Knapp method were also used in the meta-analysis [19]. Due to possible heterogeneity in patient characteristics and study design, random-effects models with the inverse variance method were used to summarize effect measures. Cochran’s Q test and the I^2^ test were used to determine the heterogeneity between studies [20]. Since the precision of both the Q and the I^2^ statistic depend on sample size, we provided another estimate, τ, which is the square root of τ^2^, the between-study variance. An obvious advantage is that its estimates do not increase systematically with the number or size of studies [21]. Publication bias was detected by funnel plot and Egger’s regression test [22]. Subgroup analyses were conducted to assess the effect of different characteristic populations or dosages on the estimates. To explore possible factors associated with the combined effect measure, we performed a meta-regression analysis using pre-set covariables. In addition, sensitivity analysis was used to assess the robustness and reliability of the results.

All above statistical analyses were carried out in R, version 4.1.3. The packages “meta” (version 5.2.0) and “metafor” (version 3.4.0) were applied to carry out the meta-analysis [23, 24]. The R code to run these operations is provided in the eMethods of the Supplement. All tests were bilateral, and the significance level was set at *P* < 0.05.

## Results

### Study and patient characteristics

**Fig. 1:**
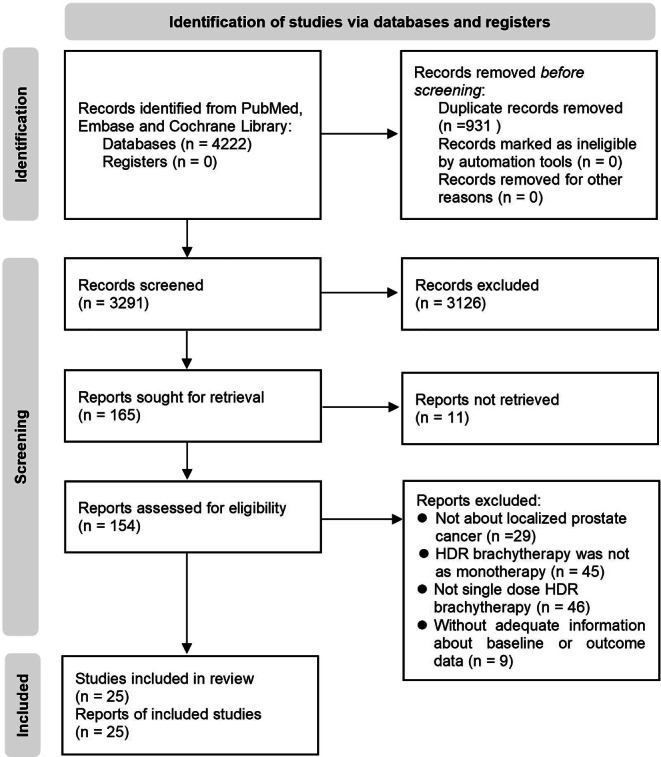
Flow diagram of systematic reviews and meta-analyses based on PRISMA. *HDR* high-dose-rate

According to the search strategy, 4222 records were identified, of which 931 were duplicate records. Twenty-five studies including 1440 patients fit the inclusion criteria for quantitative analysis after screening full-text articles and abstracts to determine eligibility [6, 7, 10, 25–46]. The PRISMA flow diagram for the recognition and selection of studies is shown in Fig. 1. There were 5 retrospective studies, 14 prospective studies, and 6 randomized trials, all of which were published between 2014 and 2022. Three studies were counted twice because they included two groups using different single-fraction HDRB schemes [6, 7, 32]. Statistically, each treatment group was treated as one study for calculation, which results in a total of 28 studies. The assessment of bias risk results for each included study are listed in eTables 2–3 and eFigure 1 of the Supplement. The characteristics of the studies included in the meta-analysis are summarized in Table 1. Dose constraints for each of the included studies are provided in eTable 4. In these studies, the dose regimens of SFHDR for patients included 19 Gy, 19.5 Gy, 20 Gy, 20.5 Gy, and 21 Gy. The patient-level characteristics of each study are shown in Table 2. In general, the median age of patients was 66.9 years (range 62–73 years) and the median follow-up was 47.5 months (range 12–75 months).

**Table 1:** Summary of primary studies

Variables	Studies (patients) or median (range) level
*Trials and studies*	
RCS	5 (317)
PCS	14 (921)
RT	6 (202)
Total studies	25 (1440)
Total groups compared	28 (1440)
Number of fractions	1
*Dose per fraction*	
19 Gy	15 (948)
19.5 Gy	1 (99)
19–21 Gy^a^	4 (164)
20 Gy	1 (33)
20.5 Gy	1 (60)
21 Gy	1 (26)
Single-dose plus focal boost	2 (110)
*HDR-BRT image guidance technique*	
TRUS	19 (1243)
TRUS + CT	2 (24)
TRUS + MRI	4 (173)
*Studies reporting bRFS*	19 (76.0%)
*Studies reporting toxic effects*	22 (88.0%)

*RCS* retrospective cohort study, *PCS* prospective cohort study, *RT* randomized trial, *TRUS* transrectal ultrasound, *CT* computed tomography, *MRI* magnetic resonance imaging

^a^The studies included 19–21 Gy dose groups or 19 Gy, 20 Gy, or 21 Gy within a group

**Table 2:** Patient characteristics of primary studies

Variables	Patient *n* (%) or median (range) level
*Total patients*	1440
*Follow-up (months)*	47.5 (12–75)
*Age (years)*	66.9 (62–73)
*Prostate volume (ml)*	35.5 (33.0–54.3)
*PSA (ng/ml)*	6.5 (4.4–14.6)
*ADT receipt*	339 (23.5)
*Clinical T stage*	
T1a–T2a	930 (64.6)
T2b–T2C	413 (28.7)
≥ T3a	97 (6.7)
*Gleason score*	
≤ 6	477 (33.1)
3 + 4	355 (24.7)
4 + 3	75 (5.2)
7^a^	479 (33.3)
≥ 8	54 (3.8)
*Risk group*	
Low risk	344 (23.9)
Intermediate risk	909 (63.1)
Low or intermediate risk^b^	16 (1.1)
High risk	171 (11.9)

^a^The studies only provided data for a Gleason score of 7 and did not further classify Gleason scores as 3 + 4 or 4 + 3

^b^The study did not report details on the number of patients regarding the low-risk and intermediate-risk groups

### Gastrointestinal toxicity

**Fig. 2:**
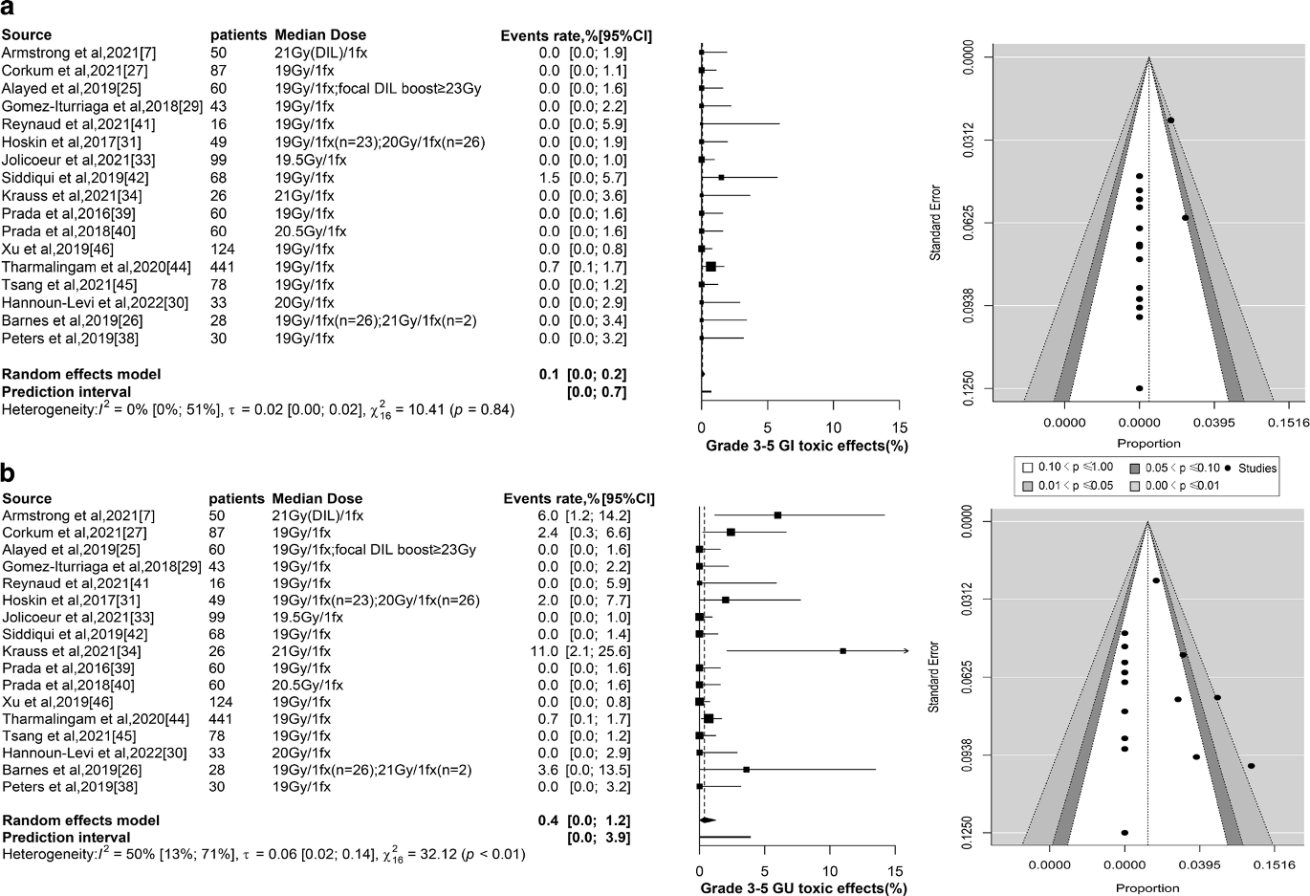
Safety. **a** Based on the weighted random-effects model with inverse variance method, severe GI toxic effects for 17 individual groups were estimated. Egger’s regression test indicated the presence of publication bias (*P* < 0.01). **b** A weighted random-effects model with inverse variance method was applied to estimate the severe GU toxic effects for 17 individual groups. Egger’s regression test indicated the absence of publication bias (*P* = 0.48). *DIL* dominant intraprostatic lesion, *fx* fraction, *GI* gastrointestinal, *GU* genitourinary

The incidence of grade 3–5 GI toxicity was very low, with only 3 cases reported, including 1 case of diarrhea and 2 cases of rectal fistula requiring colostomy [42, 44]. Based on the weighted random-effects model, the pooled cumulative occurrence was 0.1% (95% CI 0–0.2%; 95% PI 0–0.7%; Fig. 2a). The cumulative incidence of grade 2 GI toxicity was also fairly low, with an estimate of 1.6% (95% CI 0.1–4.7%; 95% PI 0–16.9%; eFigure 2a in the Supplement). Subgroup analysis showed no significant difference in the incidence of severe GI toxicity at different dosages, while statistical significance was found at grade 2 GI toxicity (*P* = 0.91 and *P* = 0.02; eFigure 3a and eFigure 3b; respectively). Note, however, that the incidence of grade 2 GI in the highest dosage subgroup was zero. eFigure 4 showed the estimated incidence of grade 3–5 and grade 2 GI toxic effects at different timepoints after receiving SFHDR, reaching the highest values at 6 months and 1 month postoperatively, respectively (0.03%, 95% CI 0–0.13%; 0.24%, 95% CI 0–1.04%).

### Genitourinary toxicity

Severe GU toxicity was also relatively rare, mainly manifested as acute hematuria, urethral stricture, and urinary retention [7, 26, 27, 31, 34, 44]. Based on the weighted random-effects model, the pooled cumulative occurrence was 0.4% (95% CI 0–1.2%; 95% PI 0–3.9%; Fig. 2b). For cumulative grade 2 GU toxicity, the pooled incidence was 17.1% (95% CI 5.4–33.5%; 95% PI 0–75.8%; eFigure 2b in the Supplement). Statistical differences were detected at different dosage subgroups for severe and grade 2 GU toxicity (*P* = 0.03 and *P* < 0.01; eFigure 3c and eFigure 3d; respectively). Again, note that the incidence of grade 2 GU in the highest dose subgroup was zero. The estimated incidence of grade 3–5 and grade 2 GU toxic events after SFHDR treatment peaked at 48 and 36 months, respectively (0.09%, 95% CI 0–0.59%; 6.31%, 95% CI 0.84–16.33%; respectively; eFigure 4). For further details of HDRB therapeutic schemes, grade 3–5 and grade 2 toxic effects were presented in Table 3 and eTable 5*.*

**Table 3:** Details of severe toxic effects and HDRB therapeutic schemes

Study	HDRB therapeutic schemes	Grade 3–5 toxic effect details
Armstrong et al. 2021 [7]	21 Gy HDRB in a single fraction, two de-escalation prescription schedules based on V_19Gy_^a^ for PTV non-boost regions	No acute grade 3 toxic effects were reported Late grade 3 toxicity: 3 patients with urethral stricture underwent surgical dilation and intermittent self-catheterization (GU)
Morton et al. 2017 [36] and Corkum et al. 2021 [27]	19 Gy HDRB in a single fraction	Acute grade 3 toxicity: 1 patient developed acute hematuria immediately after implantation requiring admission for bladder irrigation overnight (GU) Late grade 3 toxicity: 2 patients were treated with green-light laser vaporization for urinary tract obstruction or retention (GU)
Alayed et al. 2019 [25] and Alayed et al. 2021 [6]	19 Gy HDRB in a single fraction and additional focal boost to DIL at least 23 Gy	No acute grade ≥ 3 GU or GI toxic effects were observed No late grade ≥ 3 GU or GI toxic effects were observed
Gomez-Iturriaga et al. 2018 [29]	19 Gy HDRB in a single fraction	No grade 3 toxicity occurred
Hathout et al. 2019 [10] and Reynaud et al. 2021 [41]	19 Gy HDRB in a single fraction	No acute grade 3 toxicity was reported No late grade 3 toxicity was reported
Hoskin et al. 2014 [32] and Hoskin et al. 2017 [31]	19 Gy or 20 Gy HDRB in a single fraction	Acute grade 3 toxicity: 9% (2) in week 2, 4% (1) in week 4, and 9% (2) in week 12 experienced grade 3 GU toxicity Late grade 3 toxicity: 1 patient (2.6%) experienced grade 3 GU toxicity
Jolicoeur et al. 2021 [33]	19.5 Gy HDRB in a single fraction	No grade 3 toxicity was reported
Krauss et al. 2017 [35] and Siddiqui et al. 2019 [42]	19 Gy HDRB in a single fraction	No acute grade > 2 urinary or rectal toxicity was observed Late grade 3 toxicity: 1 patient developed grade 3 GI toxicity (diarrhea), which was transient and resolved after medical management
Krauss et al. 2021 [34]	21 Gy HDRB in a single fraction	Acute grade 3 toxicity: acute grade 3 urinary toxicity rates were 11%, no acute grade > 2 rectal toxic effects occurred No late grade 3 toxicity occurred
Prada et al. 2016 [39]	19 Gy HDRB in a single fraction	No grade > 2 GU or GI toxicity was observed in any patient
Prada et al. 2018 [40]	20.5 Gy HDRB in a single fraction	No grade > 2 GU or GI toxicity was observed in any patient
Xu et al. 2019 [46]	19–20 Gy HDRB in a single fraction	No grade 3 or higher acute GU or GI toxic effects were reported
Tharmalingam et al. 2020 [44]	19 Gy HDRB in a single fraction	No acute grade 3 or higher toxic effects were reported Late grade 3 toxicity: 2 patients developed late grade 3 GU toxicity, both treated with surgery for urethral stricture; 2 patients developed late grade 3 GI toxicity, both of which were rectal fistulas requiring colostomy; one of these patients had undergone pelvic chemoradiotherapy for rectal cancer
Tsang et al. 2021 [45]	19 Gy HDRB in a single fraction	No grade 3 GI and GU toxicities were found at any timepoint
Hannoun-Levi et al. 2022 [30]	20 Gy HDRB in a single fraction	No grade 3 acute toxicity or grade ≥ 2 late toxicity was observed
Barnes et al. 2019 [26]	19 Gy or 21 Gy HDRB in a single fraction	One patient experienced an acute grade 3 toxicity, experiencing urinary retention at 2 months after HDRB
Peters et al. 2019 [38]	19 Gy HDRB in a single fraction	One patient experienced acute prostate hemorrhage resulting in gross hematuria and hospital admission as a result of improper postoperative removal of an unfolded catheter, which was considered a perioperative complication in the current report

*HDRB* high-dose-rate brachytherapy, *GU* genitourinary,* GI* gastrointestinal, *PTV* planning target volume, *DIL* dominant intraprostatic lesion, *MRI* magnetic resonance imaging

^a^Percentage of volume receiving a dose of 19 Gy

### Three-year biochemical recurrence-free survival

**Fig. 3:**
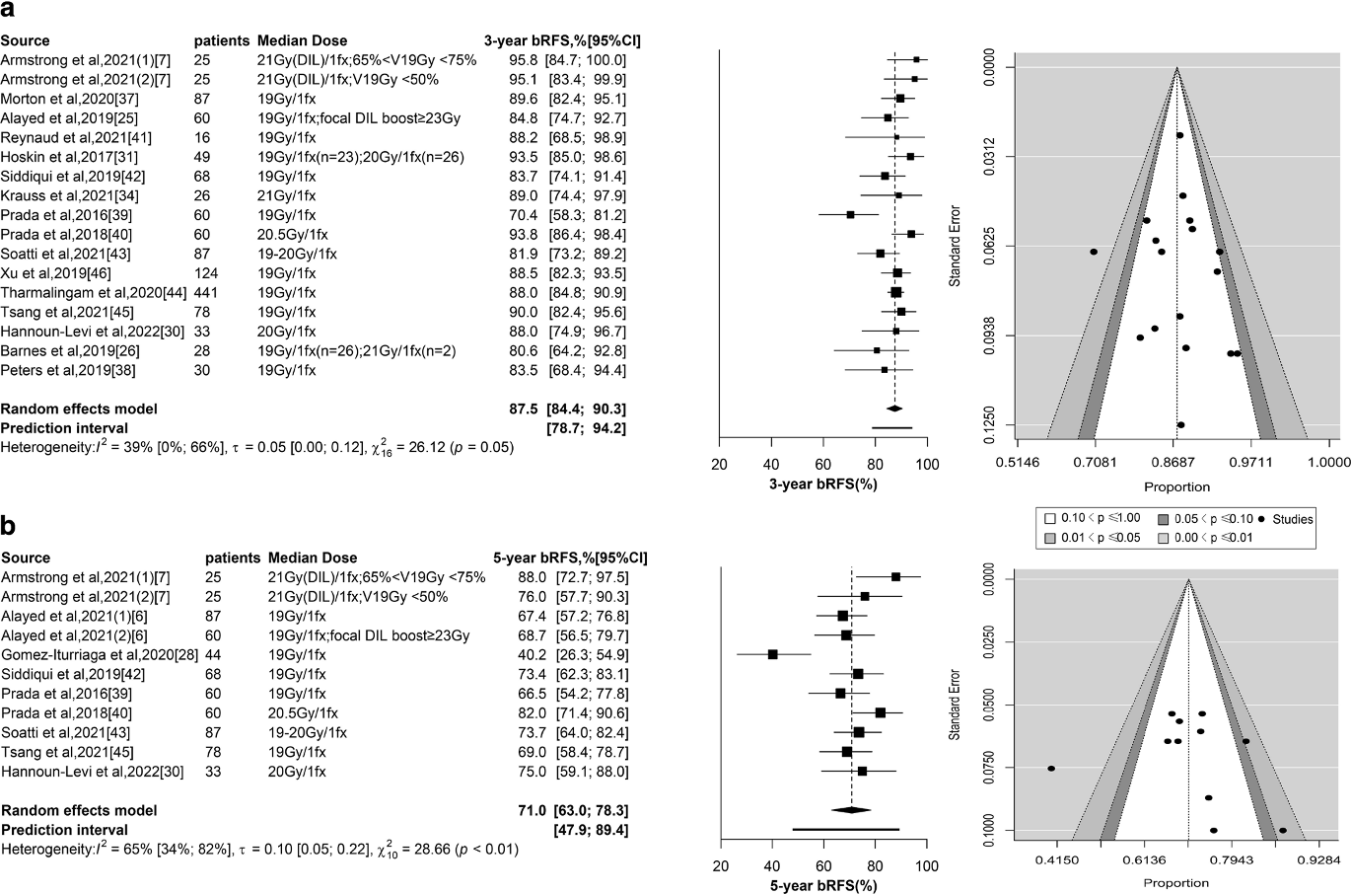
Clinical benefit. **a** Based on the weighted random-effects model with inverse variance method, 3‑year bRFS rates for 17 individual groups were estimated. Egger’s regression test indicated the absence of publication bias (*P* = 0.63). **b** A weighted random-effects model with inverse variance method was applied to estimate the 5‑year bRFS for 11 individual groups. Egger’s regression test indicated the absence of publication bias (*P* = 0.46). In the study from Armstrong et al., a single 21 Gy dose was applied to the DIL of patients and two de-escalation prescription schedules based on V_19Gy_ (percentage of volume receiving a dose of 19 Gy) for the remaining planning target volume (PTV_non-boost_). *bRFS* biochemical recurrence-free survival, *fx* fraction, *DIL* dominant intraprostatic lesion

**Fig. 4:**
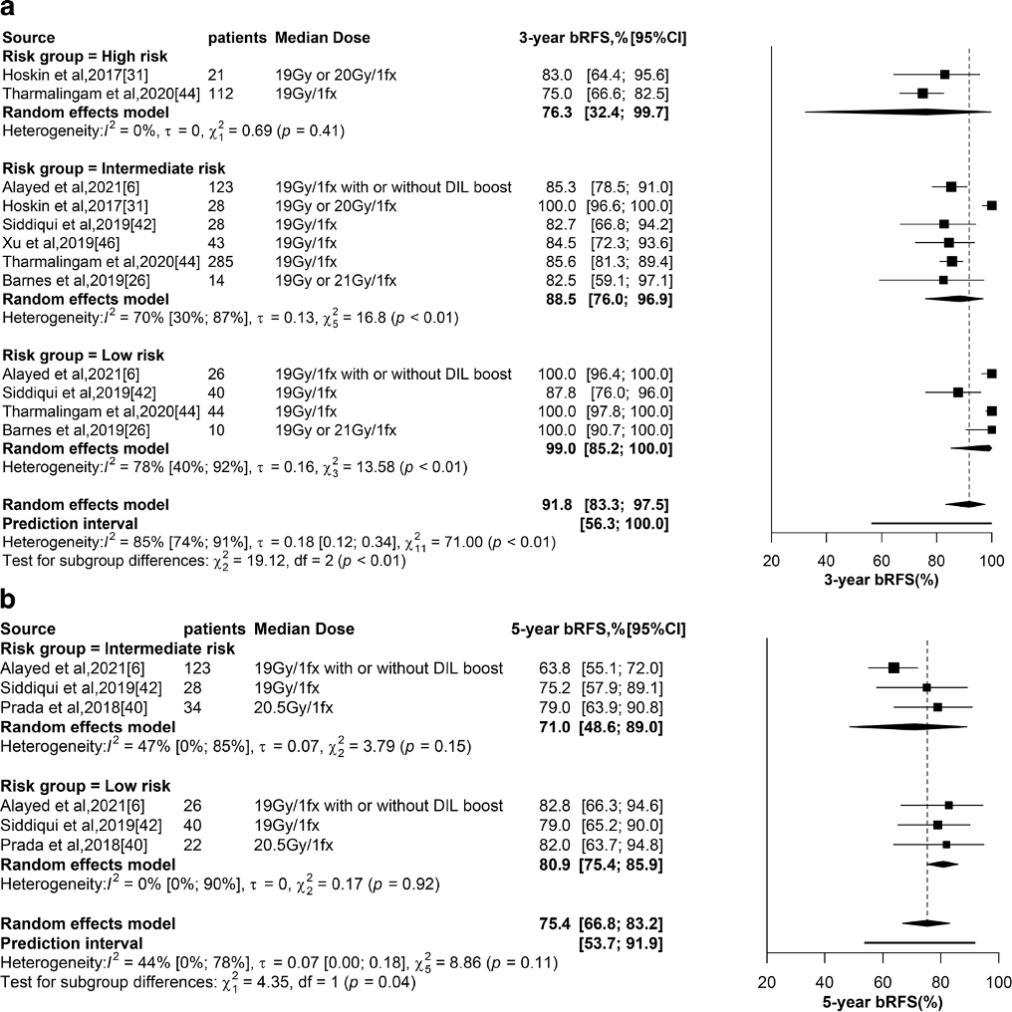
Subgroup analyses based on risk group of bRFS. **a** Subgroup analysis forest plot of 3‑year bRFS. **b** Subgroup analysis forest plot of 5‑year bRFS. Studies with unavailable data were not included in the subgroup analyses. *bRFS* biochemical recurrence-free survival, *fx* fraction, *DIL* dominant intraprostatic lesion

Data from 17 treatment groups of 16 studies were included, with 3‑year bRFS ranging from 70.4% to 95.8% [7, 25, 26, 30, 31, 34, 37–46]. Based on the weighted random-effects model, the pooled rate was 87.5% (95% CI 84.4–90.3%; 95% PI 78.7–94.2%; Fig. 3a). Sensitivity analysis showed that the estimated value remained stable by eliminating studies one by one (eTable 5a). Statistical difference was found in different risk stratifications, while it was not detected under different dosage subgroups (*P* < 0.01; *P* = 0.33; Fig. 4a and eFigure 3e; respectively). Moreover, a statistically significant difference was also found between favorable intermediate-risk (FIR) and unfavorable intermediate-risk (UIR) subgroups (*P* = 0.01). Results of multivariable meta-regression analysis showed that no significant association with 3‑year bRFS was found for three predictors (*P* = 0.08), including ADT receipt, proportion of Gleason score ≥ 7, and proportion of clinical T stage ≥ T2b (*P* = 0.12, *P* = 0.05, and *P* = 0.36, respectively; eTable 6a in the Supplement).

### Five-year bRFS

Data from 11 treatment groups of 9 studies were included, with 5‑year bRFS ranging from 40.2% (actuarial 5‑year biochemical failure rate was 59.8% from the manuscript) to 88.0% [6, 7, 28, 30, 39, 40, 42, 43, 45]. The pooled rate was 71.0% (95% CI 63.0–78.3%; 95% PI 47.9–89.4%; Fig. 3b). Sensitivity analysis showed favorable robustness of the estimate (eFigure 5b). Statistical difference was detected at different risk stratifications and was not found at dosage subgroups (*P* = 0.04; *P* = 0.06; Fig. 4b and eFigure 3f; respectively). Only one study reported 5‑year bRFS of FIR and UIR subgroups, and no statistically significant difference was detected (*P* = 0.64) [6]. Results from multivariable meta-regression analysis showed that no significant association with 5‑year bRFS was found in the three predictors (*P* = 0.51), involving ADT receipt, proportion of Gleason score ≥ 7, and the proportion of clinical T stage ≥ T2b (*P* = 0.32; *P* = 0.89; *P* = 0.88, respectively; *eTable 6b *in the Supplement).

## Discussion

The safety and efficacy of multi-fraction HDRB as monotherapy has been widely established, with studies reporting low toxicity rates and excellent bRFS rates [2, 47, 48]. However, due to the challenges of multi-fraction treatments such as resource utilization, cost control, and patient convenience, studies of SFHDR in localized prostate cancer are gradually emerging. To our knowledge, this is the first meta-analysis of the safety and efficacy associated with SFHDR for localized prostate cancer.

The results of our study provide evidence supporting the favorable tolerability of SFHDR, with cumulative severe GI and GU toxic estimates of 0.1% and 0.4%, respectively, and the corresponding grade 2 toxicity estimated at 1.6% and 17.1%, respectively. It is encouraging that a single fraction of HDRB can be comparable to fractionated HDRB, LDRB, stereotactic body radiotherapy (SBRT), and EBRT in terms of toxic effects [5, 49–51]. Prospective study results showed that the cumulative incidence of grade 2 or worse GI and GU was 6% and 33% in multi-fraction HDRB regimens [31]. A randomized study found no significant difference in GI and GU toxicity between SFHDR and LDRB [41]. The rate of ≥ grade 2 GI toxicity of SFHDR was found to be significantly lower than for fractionated SBRT (*P* < 0.05) [45]. Moreover, The CHHiP trial reported rates of 49% and 38% regarding acute ≥ grade 2 bladder and bowel toxicity after hypofractionated radiotherapy, respectively, while the crude estimates of our study remained below 20% or even lower at different timepoints after SFHDR [49]. Cumulative late ≥ grade 2 GI and GU toxicity after EBRT was 12% and 23% in the standard arm of the FLAME trial, indicating more frequent toxic events than with SFHDR [52]. In comparison, our findings suggest that the current single-fraction dosages of HDRB are acceptable, and the process of these studies is not significantly restricted by toxicity. Notably, some severe toxic effects may develop from grade 1 or 2, so early procedural intervention and close care are required to reduce the occurrence of increased toxicity. Further increase of the dose of a single fraction seems feasible, but the association between toxicity and clinical benefit should be fully considered.

Radiation-related toxicity can be prevented or mitigated in several ways. Hyaluronic acid injection through the perineum into the perirectal fat of patients could keep the rectal wall away from the radiation source and could help to avoid acute and late toxicity during the entire process, even when a single dose of 20.5 Gy HDRB was applied to the planning target volume (PTV) of the prostate [39, 40]. A recent meta-analysis evaluating seven studies showed that placement of a perirectal hydrogel spacer could significantly reduce rectal toxicity and improve bowel-related quality of life [53]. The application of these methods may facilitate further SFHDR studies under good control of radiation-related toxicity.

In terms of efficacy, a single dose of HDRB provided suboptimal biochemical control, with the estimate of 87.5% for 3‑year bRFS. Note that based on the principle of radiobiology, 19 Gy was already a not low dosage, which can achieve a similar biologically equivalent dose (BED) to 2 × 13 Gy and 3 × 10.5 Gy of HDRB for prostate cancer with an α/β ratio hypothesized to be 1.5 [8]. One prospective study demonstrated similar clinical benefits under these regimens with 4‑year bRFS approaching 90% (*P* > 0.05) [31]. Besides, SFHDR appeared to have comparable disease control effects with SBRT, given that a single 24 Gy SBRT achieved a 3-year bRFS of 86.3% and 4‑year bRFS of 77.1% [54]. In spite of this, patient selection may need to be well considered for SFHDR, with 3‑bRFS estimates of 99.0% and 5‑year bRFS estimates of 80.9% in low-risk patients, both of which were found to be statistically significant in risk stratification by bRFS (*P* < 0.05).

Nevertheless, in contrast, there was still a certain gap to other well-known experimental results. The POP-RT trial reported a 5-year BFFS of 81.2% (95% CI 71.6–87.8%) after 68 Gy in 25 fractions of prostate intensity-modulated radiotherapy (IMRT), but for high-risk patients with minimum 2 years of ADT [55]. The FLAME trial results showed that patients treated with 77 Gy in 35 fractions of EBRT acquired a 5-year bDFS of 85% (95% CI 80–89%) [52]. Besides, an estimated 5‑year failure-free survival of 84% (95% CI 80–87%) was reported from the HYPO-RT-PC study, with more than 80% intermediate-risk patients [56]. Current opinion supports the notion that most biochemical failures of SFHDR were local failures and occurred primarily at the site of initial disease [28, 37, 57]. This pattern of relapse provided researchers with a new therapeutic approach, i.e., to add a local boost to the lesion of interest based on SFHDR, although the studies failed to achieve the desired clinical benefit [6, 7, 25]. There are some possible radiobiological explanations. Because of the complex biochemical mechanism of malignant tumors, the very flexible DNA damage repair ability of tumor cells, especially cancer stem cells (CSCs), remains a concern, although the therapeutic effects can be observed at the macro level [58]. The absence of reoxygenation during SFHDR treatment may reduce the sensitivity of the tumor to radiation [59]. Moreover, due to the heterogeneity of tumors, some cancer cells with a higher α/β ratio may have relative resistance to single-fraction radiotherapy, which means that tumor cells cannot be sufficiently suppressed or killed [7].

Recently, investigators found a dose-response relationship in patients treated with SFHDR and favorable long-term disease control may be obtained by further increasing the dose of SFHDR [28, 40, 60]. The 5‑year bRFS at a dose up to 20.5 Gy reached 82.0%, better than the estimate of our study [40]. However, no significant correlation of dosage with bRFS was found for 19–21 Gy, possibly due to the narrow dose range that was not powered to detect the presence of a difference. Clinical trials at a dose up to 23 Gy and 25 Gy (NCT03424850) are underway, and the mature results will help us to better understand the toxicity and efficacy of SFHDR.

Although we conducted this meta-analysis strictly and carefully, some limitations should be recognized. First, due to the inconsistencies of control groups or the absence of a control group, our study only combined the incidence of a single treatment group of included studies, so a cautious attitude should be taken to evaluating the outcome. Second, our median follow-up was no more than 50 months, and the long-term follow-up data are still limited. Besides, as data from several studies are unavailable, the reliability of our subgroup analyses and meta-regression models needs to be further validated by including larger-scale studies.

## Conclusion

Overall, SFHDR is well tolerated and associated with suboptimal clinical benefit in patients with localized prostate cancer. High-quality prospective studies of SFHDR are necessary to verify its safety and efficacy.

## Supplementary Information

eFigures 1–5. Supplemental figures include the risk of bias graph, estimates of grade 2 toxic effects, subgroup analyses, the occurrence of toxic effects at different time points, and sensitivity analyses, respectively.

eTables 1–6 and eMethods. Supplemental tables include PICOS inclusion criteria, the risk of bias assessment for randomized design and non-randomized studies, details of the included studies, and results of meta regression analyses, respectively

## Abbreviations

ADT: Androgen deprivation therapy
BF: Biochemical failure
bRFS: Biochemical recurrence-free survival
DIL: Dominant intraprostatic lesion
EBRT: External-beam radiation therapy
GI: Gastrointestinal
GU: Genitourinary
HDRB: High-dose-rate brachytherapy
LDRB: Low-dose-rate brachytherapy
MINORS: Methodological index for non-randomized studies
PRISMA: Preferred Reporting Items for Systematic Reviews and Meta-Analyses
PROSPERO: Prospective Register of Systematic Reviews
PSA: Prostate-specific antigen
PTV: Planning target volume
REML: Restricted maximum-likelihood estimator
RoB 2: Revised Cochrane Risk-of-bias tool for randomized trials
SFHDR: Single-fraction high-dose-rate brachytherapy

## References

[1] Crook J, Marban M, Batchelar D (2020). HDR prostate brachytherapy. Semin Radiat Oncol.

[2] Strouthos I, Tselis N, Chatzikonstantinou G (2018). High dose rate brachytherapy as monotherapy for localised prostate cancer. Radiother Oncol.

[3] Nagore G, Lopez Guerra JL, Krumina E (2018). High dose rate brachytherapy for prostate cancer: a prospective toxicity evaluation of a one day schedule including two 13.5 Gy fractions. Radiother Oncol.

[4] Patel S, Demanes DJ, Ragab O (2017). High-dose-rate brachytherapy monotherapy without androgen deprivation therapy for intermediate-risk prostate cancer. Brachytherapy.

[5] Yamazaki H, Masui K, Suzuki G (2018). Comparison of three moderate fractionated schedules employed in high-dose-rate brachytherapy monotherapy for clinically localized prostate cancer. Radiother Oncol.

[6] Alayed Y, Loblaw A, McGuffin M (2021). Single-fraction HDR brachytherapy as monotherapy in low and intermediate risk prostate cancer: outcomes from two clinical trials with and without an MRI-guided boost. Radiother Oncol.

[7] Armstrong S, Brown S, Stancliffe M (2021). Single dose high-dose-rate brachytherapy with focal dose escalation for prostate cancer: mature results of a phase 2 clinical trial. Radiother Oncol.

[8] Brenner DJ, Martinez AA, Edmundson GK, Mitchell C, Thames HD, Armour EP (2002). Direct evidence that prostate tumors show high sensitivity to fractionation (low alpha/beta ratio), similar to late-responding normal tissue. Int J Radiat Oncol Biol Phys.

[9] Fowler J, Chappell R, Ritter M (2001). Is alpha/beta for prostate tumors really low?. Int J Radiat Oncol Biol Phys.

[10] Hathout L, Mahmoud O, Wang Y (2019). A phase 2 randomized pilot study comparing high-dose-rate brachytherapy and low-dose-rate brachytherapy as monotherapy in localized prostate cancer. Adv Radiat Oncol.

[11] Mendez LC, Morton GC (2018). High dose-rate brachytherapy in the treatment of prostate cancer. Transl Androl Urol.

[12] Dess RT, Soni PD, Jackson WC (2019). The current state of randomized clinical trial evidence for prostate brachytherapy. Urol Oncol.

[13] Moher D, Liberati A, Tetzlaff J, Altman DG (2009). Preferred reporting items for systematic reviews and meta-analyses: the PRISMA statement. BMJ.

[14] Zhang X, Geng P, Zhang T, Lu Q, Gao P, Mei J (2020). Aceso: PICO-guided evidence summarization on medical literature. IEEE J Biomed Health Inform.

[15] Frandsen TF, Bruun Nielsen MF, Lindhardt CL, Eriksen MB (2020). Using the full PICO model as a search tool for systematic reviews resulted in lower recall for some PICO elements. J Clin Epidemiol.

[16] Sterne JAC, Savović J, Page MJ (2019). RoB 2: a revised tool for assessing risk of bias in randomised trials. BMJ.

[17] Slim K, Nini E, Forestier D, Kwiatkowski F, Panis Y, Chipponi J (2003). Methodological index for non-randomized studies (minors): development and validation of a new instrument. ANZ J Surg.

[18] Roach M, Hanks G, Thames H (2006). Defining biochemical failure following radiotherapy with or without hormonal therapy in men with clinically localized prostate cancer: recommendations of the RTOG-ASTRO Phoenix Consensus Conference. Int J Radiat Oncol Biol Phys.

[19] Langan D, Higgins JPT, Jackson D (2019). A comparison of heterogeneity variance estimators in simulated random-effects meta-analyses. Res Syn Meth.

[20] Higgins JP, Thompson SG (2002). Quantifying heterogeneity in a meta-analysis. Statist Med.

[21] Rücker G, Schwarzer G, Carpenter JR, Schumacher M (2008). Undue reliance on I(2) in assessing heterogeneity may mislead. BMC Med Res Methodol.

[22] Egger M, Davey Smith G, Schneider M, Minder C (1997). Bias in meta-analysis detected by a simple, graphical test. BMJ.

[23] Balduzzi S, Rücker G, Schwarzer G (2019). How to perform a meta-analysis with R: a practical tutorial. Evid Based Ment Health.

[24] Viechtbauer W (2010). Conducting meta-analyses in R with the metafor package. J Stat Soft.

[25] Alayed Y, D’Alimonte L, Helou J (2019). MRI assisted focal boost integrated with HDR monotherapy study in low and intermediate risk prostate cancer (MARS): results from a phase II clinical trial. Radiother Oncol.

[26] Barnes JM, Gabani P, Sanders M (2019). Single fraction high-dose-rate brachytherapy as monotherapy for low and intermediate risk prostate cancer: toxicities and early outcomes from a single institutional experience. J Contemp Brachytherapy.

[27] Corkum M, Loblaw A, Hasan Y (2021). Prostate high dose-rate brachytherapy as monotherapy for prostate cancer: late toxicity and patient reported outcomes from a randomized phase II clinical trial. Radiother Oncol.

[28] Gomez-Iturriaga A, Buchser D, Mayrata E (2020). Pattern of relapse and dosimetric analysis of a single dose 19 Gy HDR-brachytherapy phase II trial. Radiother Oncol.

[29] Gomez-Iturriaga A, Casquero F, Pijoan JI (2018). Health-related-quality-of-life and toxicity after single fraction 19 Gy high-dose-rate prostate brachytherapy: phase II trial. Radiother Oncol.

[30] Hannoun-Levi JM, Chand-Fouche ME, Pace-Loscos T (2022). Single fraction of HDR brachytherapy for prostate cancer: results of the siFEPI phase II prospective trial. Clin Transl Radiat Oncol.

[31] Hoskin P, Rojas A, Ostler P, Hughes R, Alonzi R, Lowe G (2017). Single-dose high-dose-rate brachytherapy compared to two and three fractions for locally advanced prostate cancer. Radiother Oncol.

[32] Hoskin P, Rojas A, Ostler P (2014). High-dose-rate brachytherapy alone given as two or one fraction to patients for locally advanced prostate cancer: acute toxicity. Radiother Oncol.

[33] Jolicoeur M, Derashodian T, Nguyen-Huynh T (2021). PP-0121 HDR brachytherapy as monotherapy for prostate cancer: early toxicity of a randomized phase II trial. Radiother Oncol.

[34] Krauss D, Ye H, Sebastian E (2021). 21 Gy single fraction high dose rate brachytherapy for early stage prostate cancer: early outcomes from a single institution prospective clinical trial. Brachytherapy.

[35] Krauss DJ, Ye H, Martinez AA (2017). Favorable preliminary outcomes for men with low- and intermediate-risk prostate cancer treated with 19-Gy single-fraction high-dose-rate brachytherapy. Int J Radiat Oncol Biol Phys.

[36] Morton G, Chung HT, McGuffin M (2017). Prostate high dose-rate brachytherapy as monotherapy for low and intermediate risk prostate cancer: early toxicity and quality-of life results from a randomized phase II clinical trial of one fraction of 19 Gy or two fractions of 13.5 Gy. Radiother Oncol.

[37] Morton G, McGuffin M, Chung HT (2020). Prostate high dose-rate brachytherapy as monotherapy for low and intermediate risk prostate cancer: efficacy results from a randomized phase II clinical trial of one fraction of 19 Gy or two fractions of 13.5 Gy. Radiother Oncol.

[38] Peters M, van Son MJ, Moerland MA (2019). MRI-guided ultrafocal HDR brachytherapy for localized prostate cancer: median 4-year results of a feasibility study. Int J Radiat Oncol Biol Phys.

[39] Prada PJ, Cardenal J, Blanco AG (2016). High-dose-rate interstitial brachytherapy as monotherapy in one fraction for the treatment of favorable stage prostate cancer: toxicity and long-term biochemical results. Radiother Oncol.

[40] Prada PJ, Ferri M, Cardenal J (2018). High-dose-rate interstitial brachytherapy as monotherapy in one fraction of 20.5 Gy for the treatment of localized prostate cancer: toxicity and 6-year biochemical results. Brachytherapy.

[41] Reynaud T, Hathout L, Carignan D (2021). PSA outcomes and late toxicity of single-fraction HDR brachytherapy and LDR brachytherapy as monotherapy in localized prostate cancer: a phase 2 randomized pilot study. Brachytherapy.

[42] Siddiqui ZA, Gustafson GS, Ye H (2019). Five-year outcomes of a single-institution prospective trial of 19-Gy single-fraction high-dose-rate brachytherapy for low- and intermediate-risk prostate cancer. Int J Radiat Oncol Biol Phys.

[43] Soatti CP, Delishaj D, D’Amico R (2021). High-dose-rate brachytherapy as monotherapy for localized prostate cancer using three different doses—14 years of single-centre experience. J Contemp Brachytherapy.

[44] Tharmalingam H, Tsang Y, Ostler P (2020). Single dose high-dose rate (HDR) brachytherapy (BT) as monotherapy for localised prostate cancer: early results of a UK national cohort study. Radiother Oncol.

[45] Tsang YM, Tharmalingam H, Belessiotis-Richards K (2021). Ultra-hypofractionated radiotherapy for low- and intermediate risk prostate cancer: High-dose-rate brachytherapy vs stereotactic ablative radiotherapy. Radiother Oncol.

[46] Xu MJ, Chen KS, Chang AJ (2019). Single-fraction brachytherapy as monotherapy for early-stage prostate cancer: the UCSF experience. Brachytherapy.

[47] Behmueller M, Tselis N, Zamboglou N (2021). High-dose-rate brachytherapy as monotherapy for low- and intermediate-risk prostate cancer. Oncological outcomes after a median 15-year follow-up. Front Oncol.

[48] Yamazaki H, Masui K, Suzuki G (2019). High-dose-rate brachytherapy monotherapy versus low-dose-rate brachytherapy with or without external beam radiotherapy for clinically localized prostate cancer. Radiother Oncol.

[49] Dearnaley D, Syndikus I, Mossop H (2016). Conventional versus hypofractionated high-dose intensity-modulated radiotherapy for prostate cancer: 5-year outcomes of the randomised, non-inferiority, phase 3 CHHiP trial. Lancet Oncol.

[50] Kishan AU, Dang A, Katz AJ (2019). Long-term outcomes of stereotactic body radiotherapy for low-risk and intermediate-risk prostate cancer. JAMA Netw Open.

[51] Vuolukka K, Auvinen P, Palmgren JE, Voutilainen T, Aaltomaa S, Kataja V (2019). Long-term efficacy and urological toxicity of low-dose-rate brachytherapy (LDR-BT) as monotherapy in localized prostate cancer. Brachytherapy.

[52] Kerkmeijer LGW, Groen VH, Pos FJ (2021). Focal boost to the Intraprostatic tumor in external beam radiotherapy for patients with localized prostate cancer: results from the FLAME randomized phase III trial. J Clin Oncol.

[53] Miller LE, Efstathiou JA, Bhattacharyya SK, Payne HA, Woodward E, Pinkawa M (2020). Association of the placement of a perirectal hydrogel spacer with the clinical outcomes of men receiving radiotherapy for prostate cancer: a systematic review and meta-analysis. JAMA Netw Open.

[54] Greco C, Pares O, Pimentel N (2021). Safety and efficacy of virtual prostatectomy with single-dose radiotherapy in patients with intermediate-risk prostate cancer: results from the PROSINT phase 2 randomized clinical trial. JAMA Oncol.

[55] Murthy V, Maitre P, Kannan S (2021). Prostate-only versus whole-pelvic radiation therapy in high-risk and very high-risk prostate cancer (POP-RT): outcomes from phase III randomized controlled trial. J Clin Oncol.

[56] Widmark A, Gunnlaugsson A, Beckman L (2019). Ultra-hypofractionated versus conventionally fractionated radiotherapy for prostate cancer: 5-year outcomes of the HYPO-RT-PC randomised, non-inferiority, phase 3 trial. Lancet.

[57] Mendez LC, Ravi A, Chung H (2018). Pattern of relapse and dose received by the recurrent intraprostatic nodule in low- to intermediate-risk prostate cancer treated with single fraction 19 Gy high-dose-rate brachytherapy. Brachytherapy.

[58] Pajonk F, Vlashi E, McBride WH (2010). Radiation resistance of cancer stem cells: the 4 R’s of radiobiology revisited. Stem Cells.

[59] Supiot S, Rousseau C, Dore M (2019). Reoxygenation during radiotherapy in intermediate-risk prostate cancer. Radiother Oncol.

[60] Greco C, Zelefsky MJ, Lovelock M (2011). Predictors of local control after single-dose stereotactic image-guided intensity-modulated radiotherapy for extracranial metastases. Int J Radiat Oncol Biol Phys.

