# Supplementary material for: Safety and efficacy associated with single-fraction high-dose-rate brachytherapy in localized prostate cancer: a systematic review and meta-analysis
Source: Strahlenther Onkol. 2023 Apr 24;199(6):525–35. doi: 10.1007/s00066-023-02063-z (PMC10212877; doi:10.1007/s00066-023-02063-z)
Supplement: Supplementary file 1 — eFigures 1–5. Supplemental figures include the risk of bias graph, estimates of grade 2 toxic effects, subgroup analyses, the occurrence of toxic effects at different time points, and sensitivity analyses, respectively. [file 66_2023_2063_MOESM1_ESM.docx]

**Supplemental Figures Contents**

**[eFigure 1.](#_Toc32033)** [Risk of bias graph performed with Cochrane risk of bias tool (RoB2)](#_Toc32033)

**[eFigure 2.](#_Toc21204)** [Estimates of Grade 2 toxic effects](#_Toc21204)

**[eFigure 2a.](#_Toc17461)** [Estimate of of grade 2 GI toxic effects](#_Toc17461)

**[eFigure 2b.](#_Toc20886)** [Estimate of of grade 2 GU toxic effects](#_Toc20886)

**[eFigure 3.](#_Toc6678)** [Subgroup analyses based on dose size](#_Toc6678)

**[eFigure 3a.](#_Toc28439)** [Subgroup analysis of severe GI toxic effects](#_Toc28439)

**[eFigure 3b.](#_Toc9030)** [Subgroup analysis of grade 2 GI toxic effects](#_Toc9030)

**[eFigure 3c.](#_Toc31927)** [Subgroup analysis of severe GU toxic effects](#_Toc31927)

**[eFigure 3d.](#_Toc7695)** [Subgroup analysis of grade 2 GU toxic effects](#_Toc7695)

**[eFigure 3e.](#_Toc17162)** [Subgroup analysis of 3-year bRFS](#_Toc17162)

**[eFigure 3f.](#_Toc30721)** [Subgroup analysis of 5-year bRFS](#_Toc30721)

**[eFigure 4.](#_Toc28824)** [Occurrence of toxic effects at different time points](#_Toc28824)

**[eFigure 4a.](#_Toc30418)** [Occurrence of severe GU and GI toxic effects at different time points](#_Toc30418)

**[eFigure 4b.](#_Toc12502)** [Occurrence of grade 2 GU and GI toxic effects at different time points](#_Toc12502)

**[eFigure 5.](#_Toc1699)** [Sensitivity analyses](#_Toc1699)

**[eFigure 5a.](#_Toc31709)** [Sensitivity analysis of studies related to 3-year bRFS](#_Toc31709)

**[eFigure 5b.](#_Toc8979)** [Sensitivity analysis of studies related to 5-year bRFS](#_Toc8979)

**
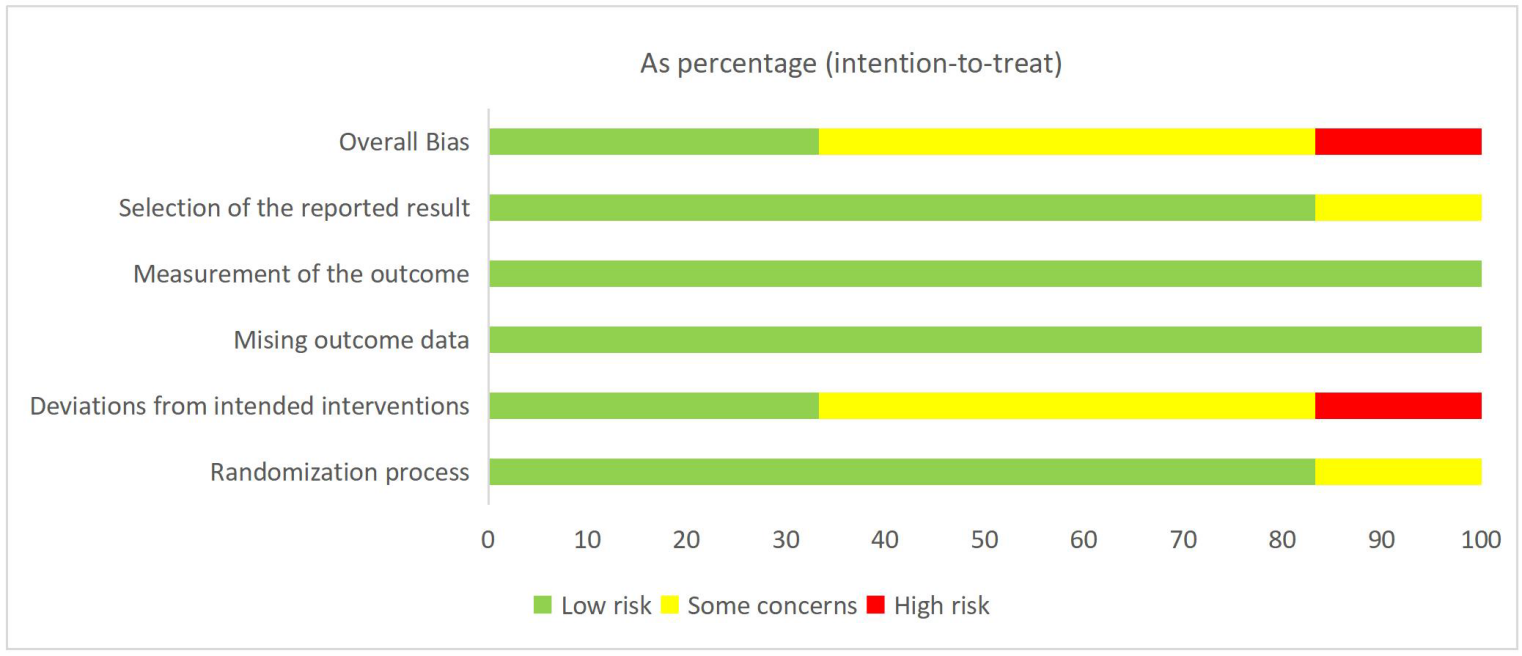
**

**eFigure 1. Risk of bias graph performed with Cochrane risk of bias tool (RoB2)**

**eFigure 2. Estimates of Grade 2 toxic effects**


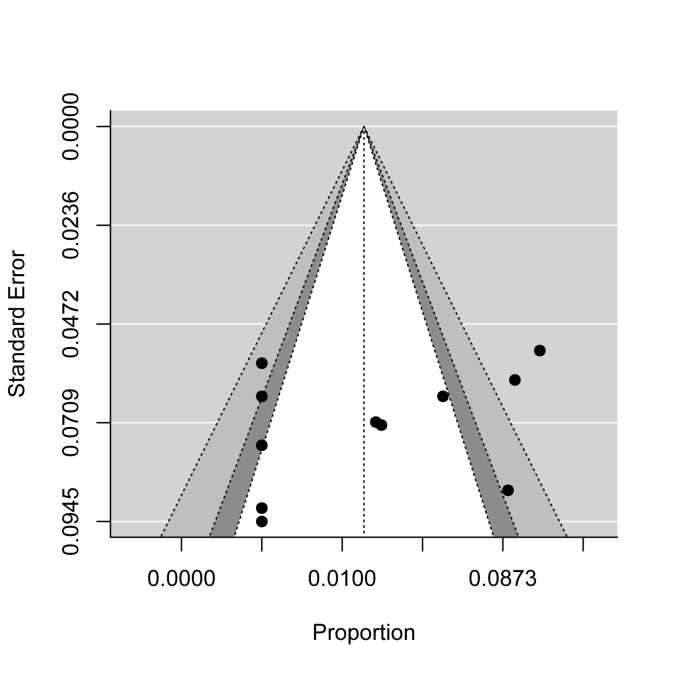

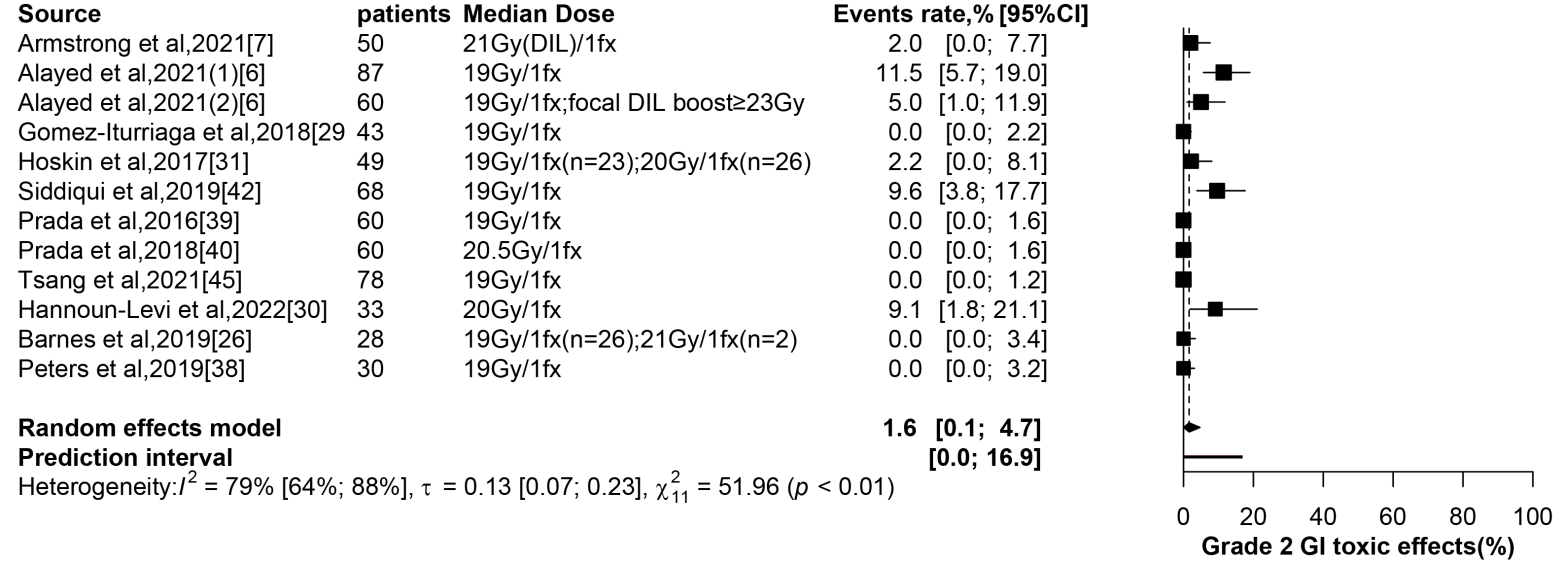


**eFigure 2a. Estimate of of grade 2 GI toxic effects**


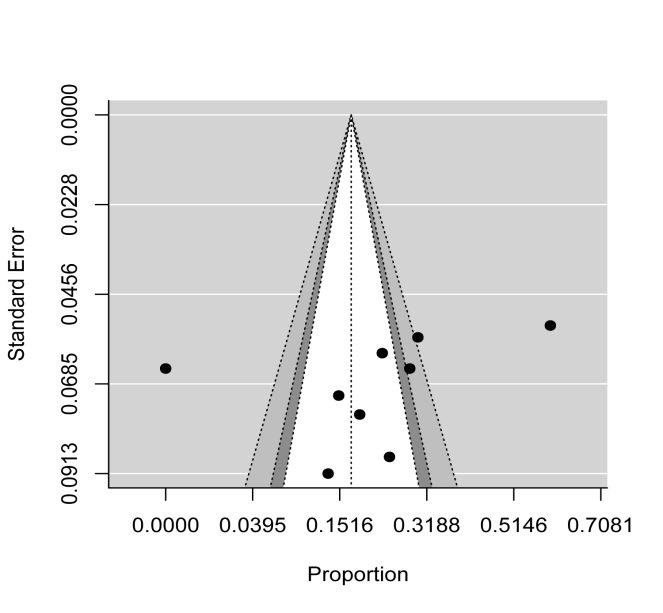

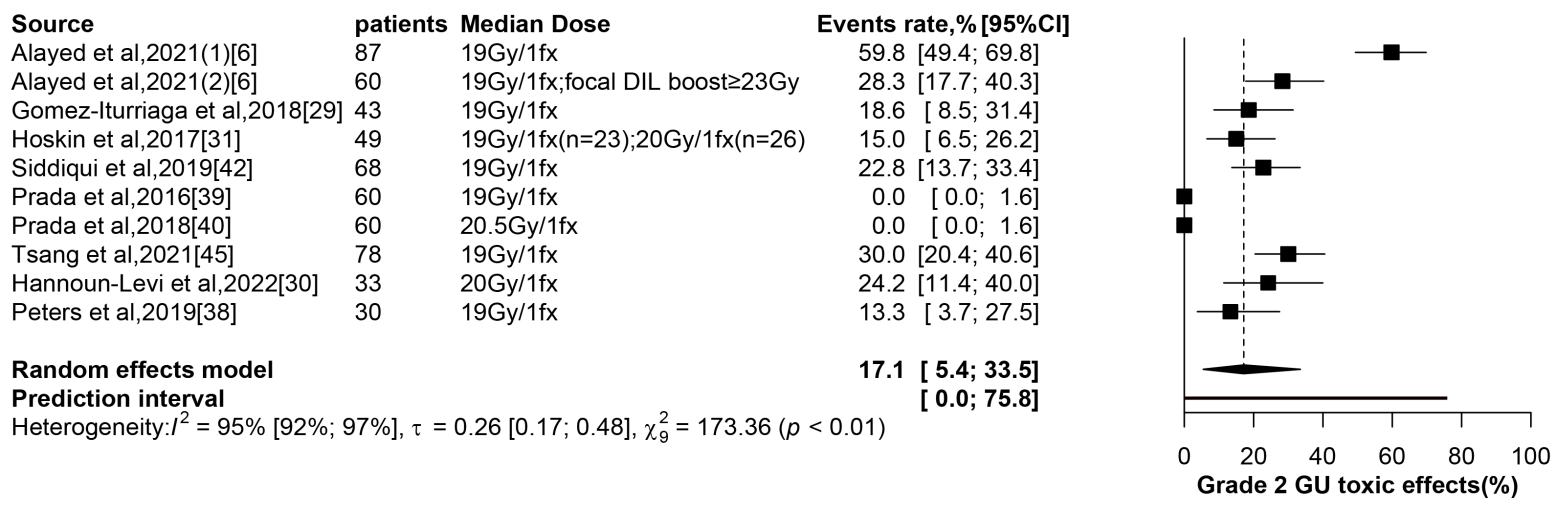


**eFigure 2b. Estimate of of grade 2 GU toxic effects**

**eFigure 3. Subgroup analyses based on dose size**

**
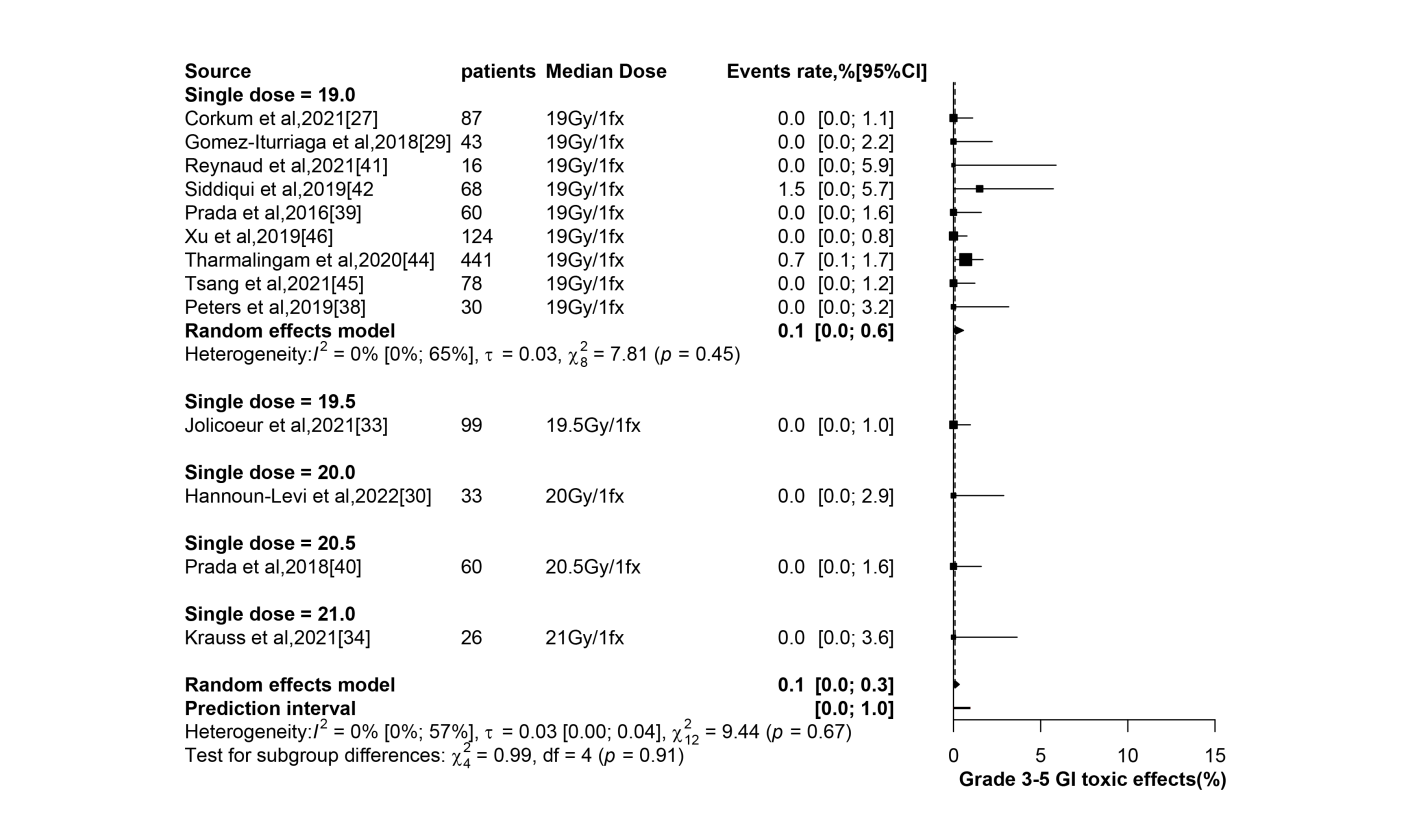
**

**eFigure 3a. Subgroup analysis of severe GI toxic effects**

**
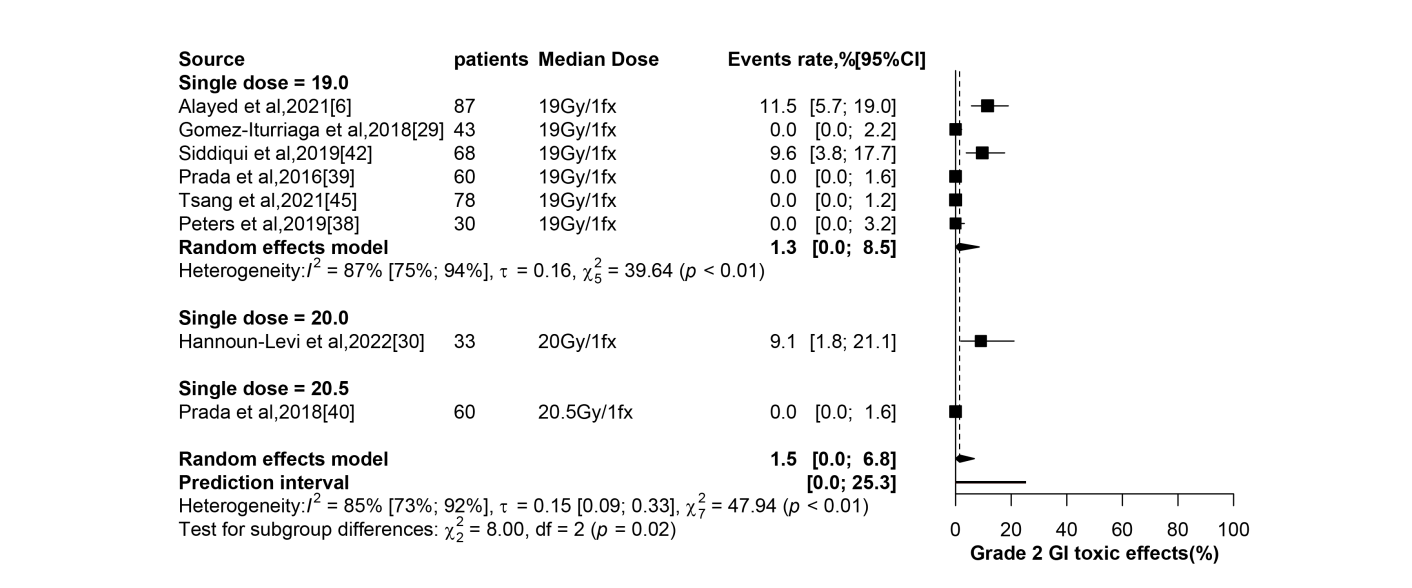
**

**eFigure 3b. Subgroup analysis of grade 2 GI toxic effects**

**
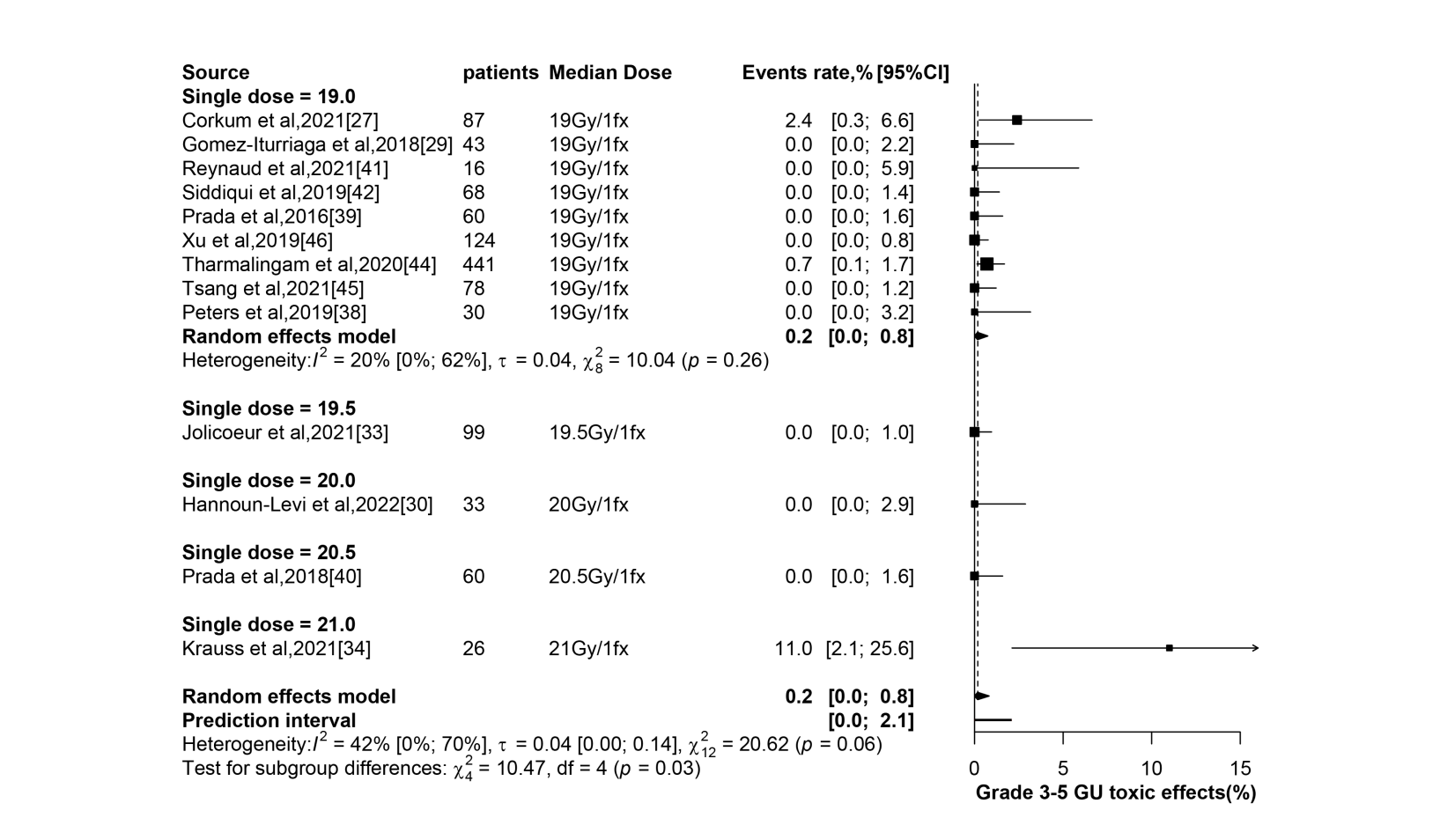
**

**eFigure 3c. Subgroup analysis of severe GU toxic effects**

**
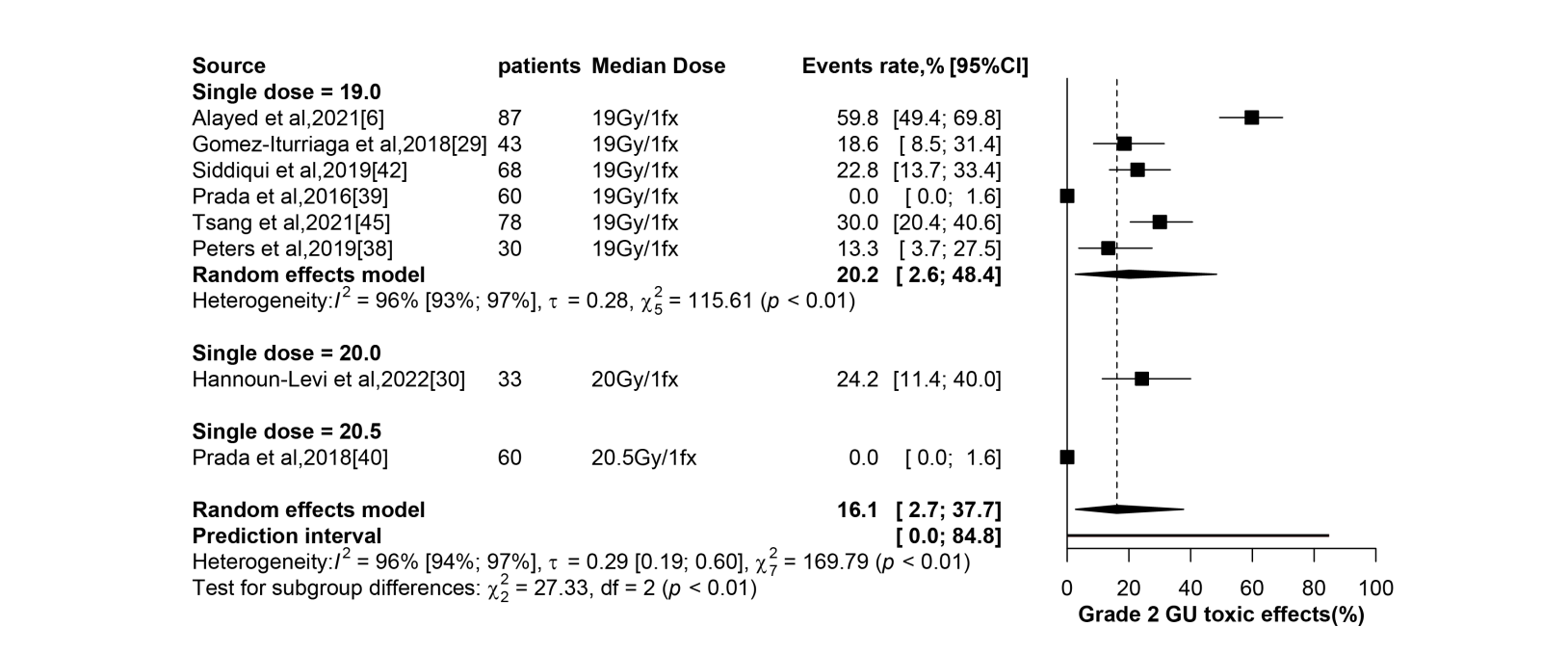
**

**eFigure 3d. Subgroup analysis of grade 2 GU toxic effects**

**
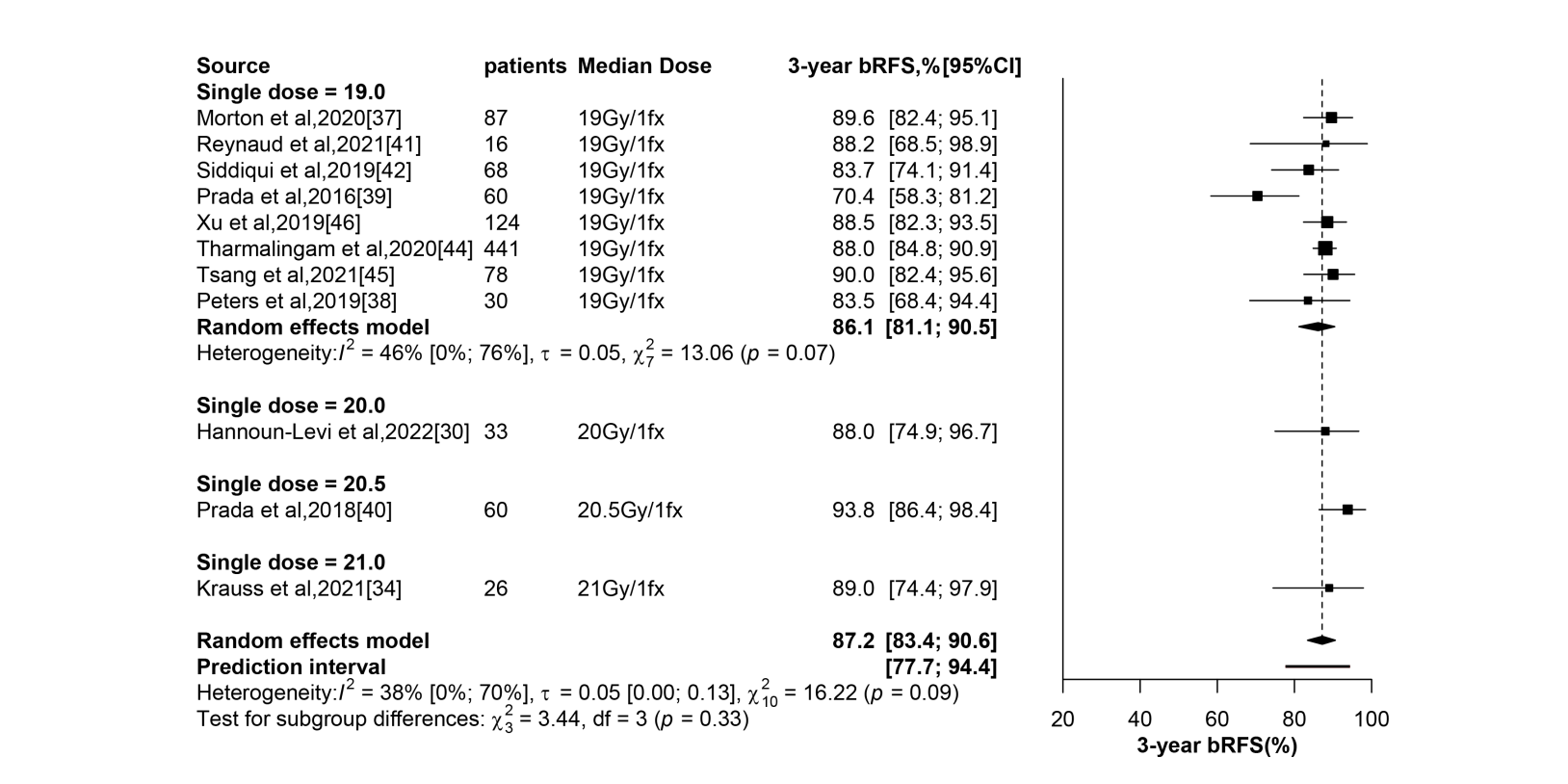
**

**eFigure 3e. Subgroup analysis of 3-year bRFS**

**
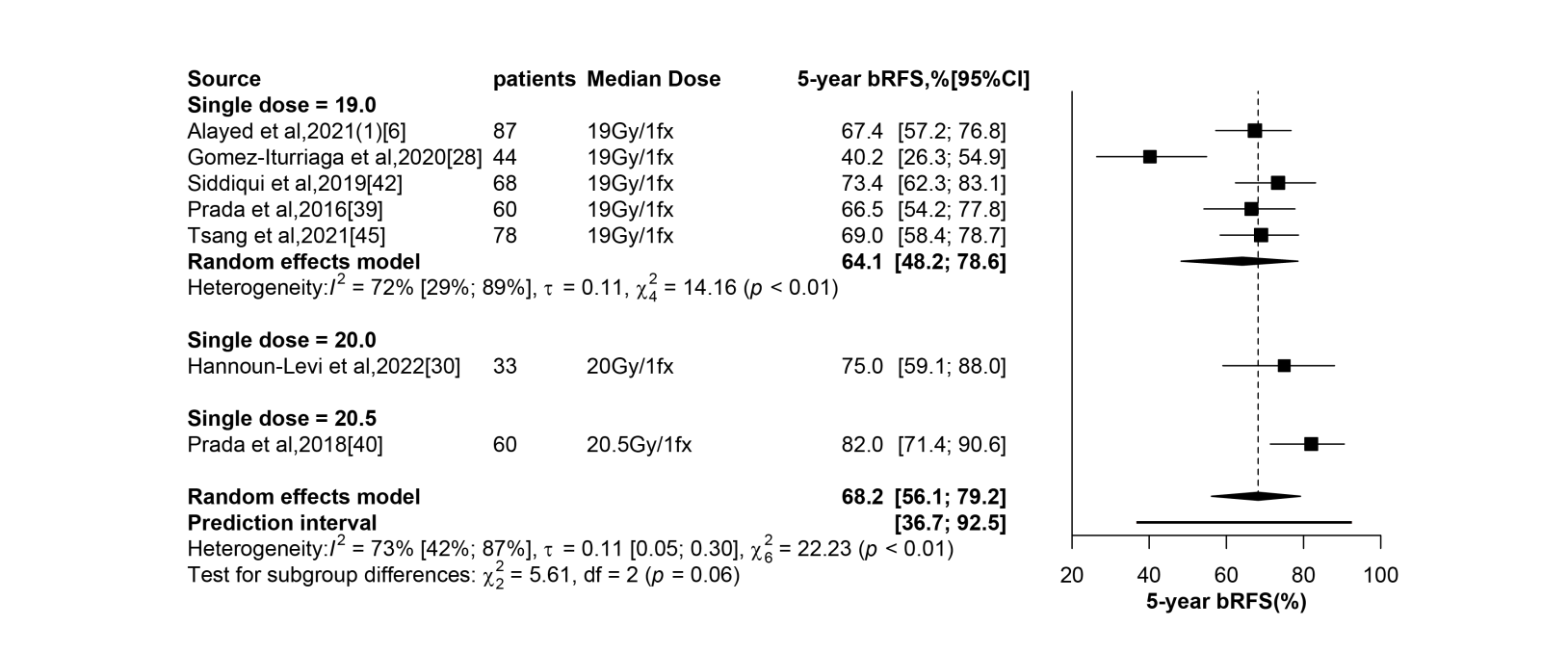
**

**eFigure 3f. Subgroup analysis of 5-year bRFS**

**eFigure 4. Occurrence of toxic effects at different time points**

**
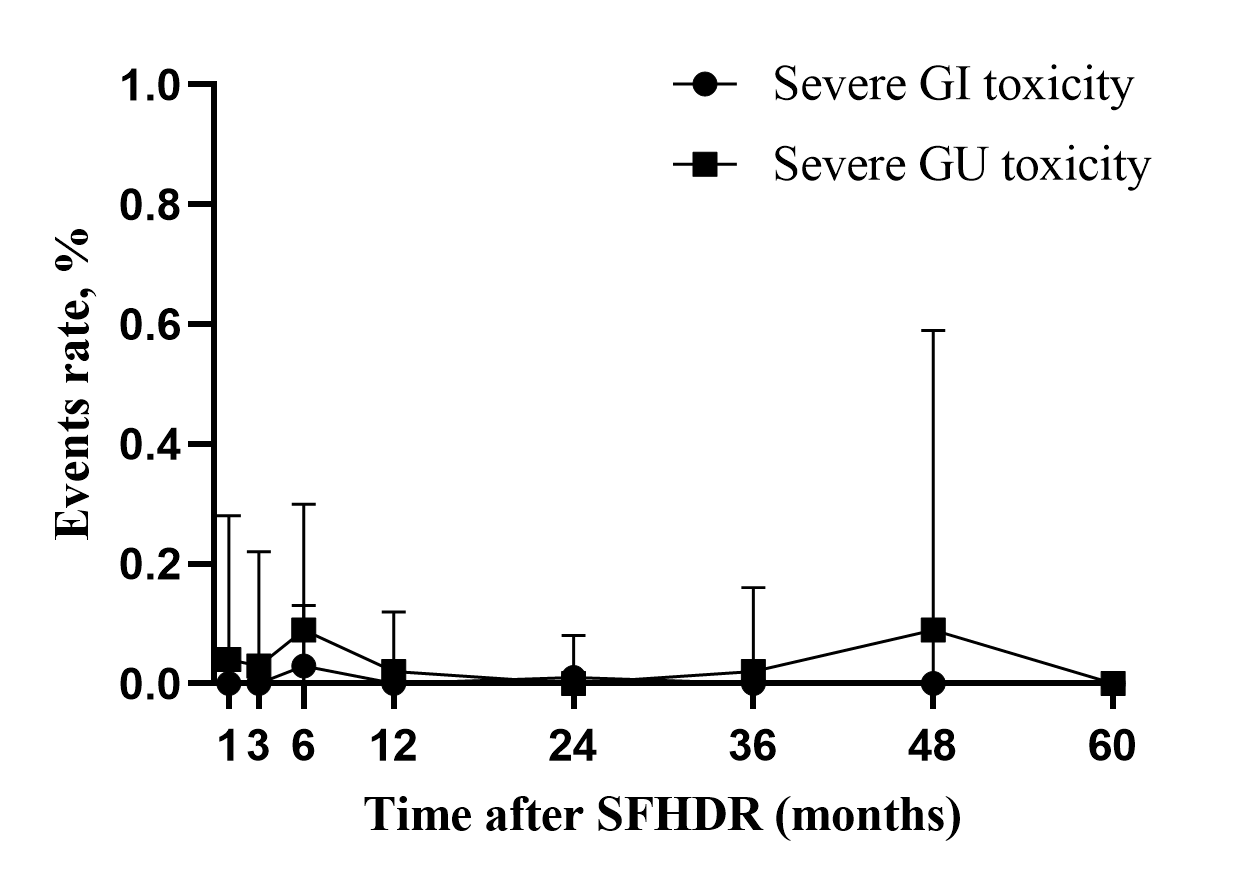
**

**eFigure 4a. Occurrence of severe GU and GI toxic effects at different time points**

**
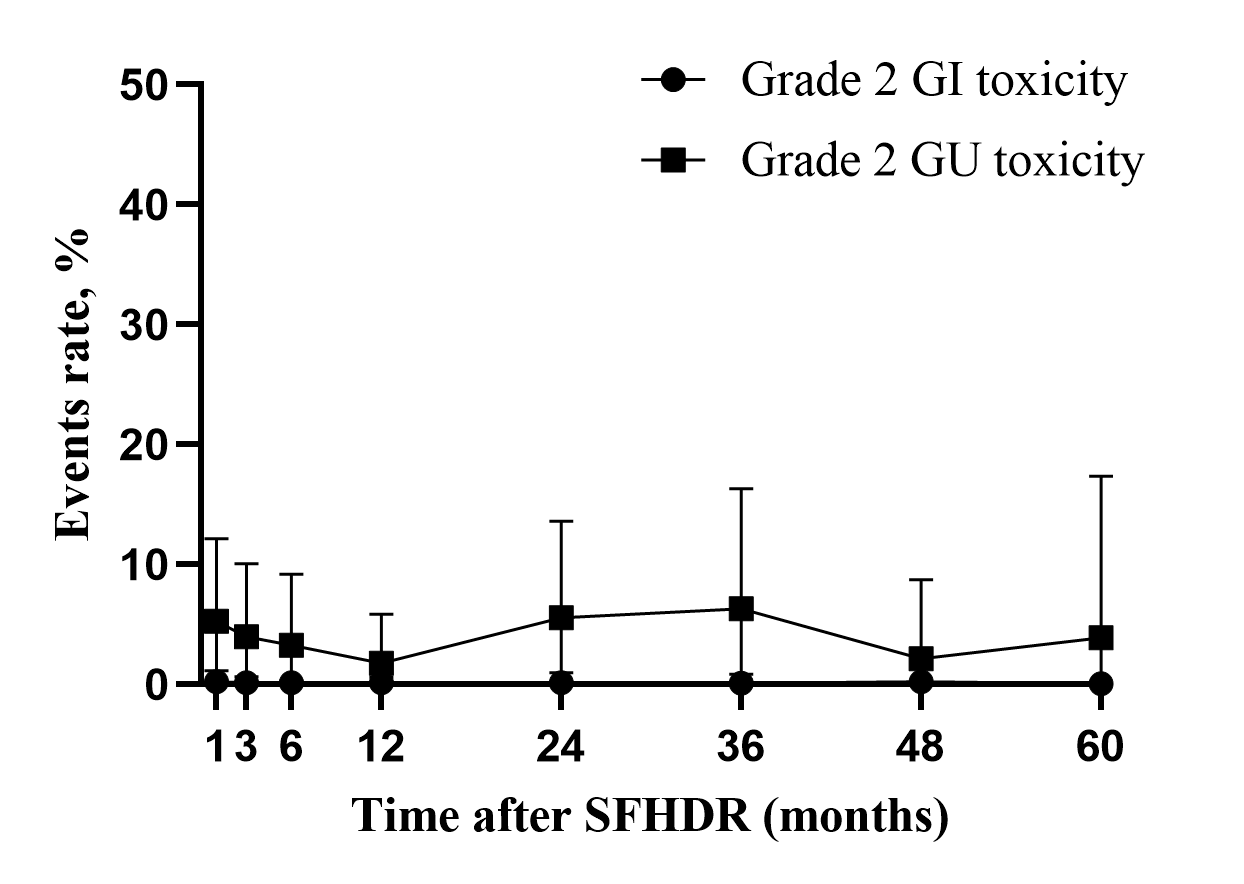
**

**eFigure 4b. Occurrence of grade 2 GU and GI toxic effects at different time points**

**eFigure 5. Sensitivity analyses**

**
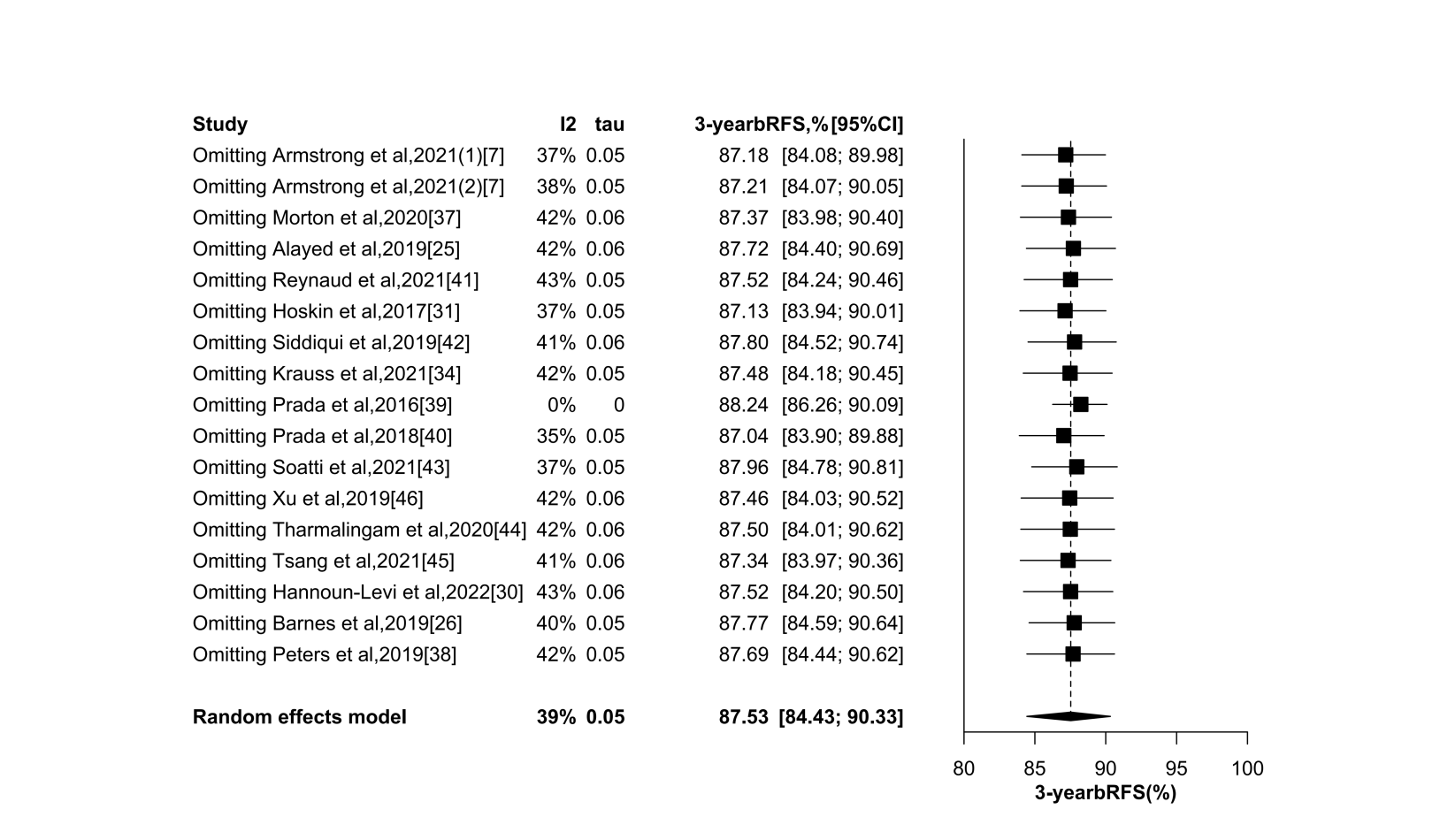
**

**eFigure 5a. Sensitivity analysis of studies related to 3-year bRFS**

**
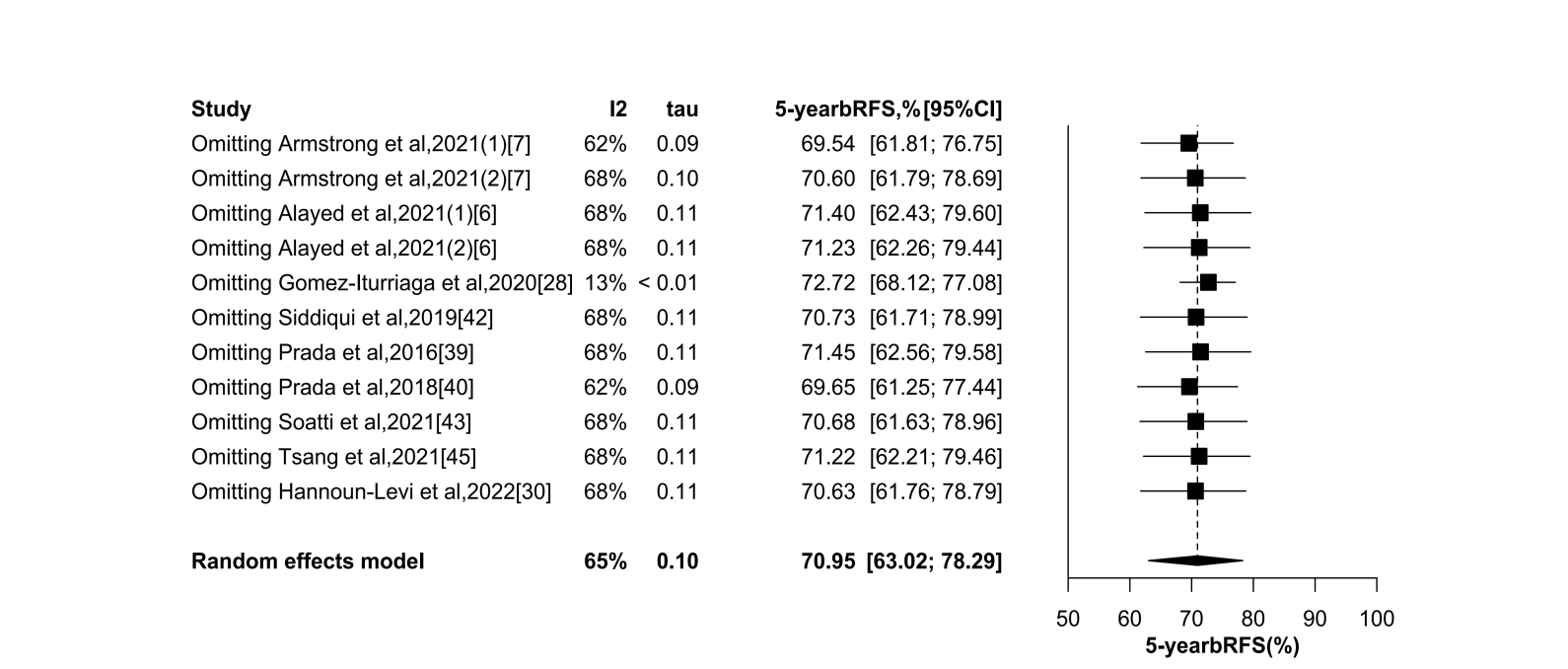
**

**eFigure 5b. Sensitivity analysis of studies related to 5-year bRFS**
