# Supplementary material for: Safety and efficacy associated with single-fraction high-dose-rate brachytherapy in localized prostate cancer: a systematic review and meta-analysis
Source: Strahlenther Onkol. 2023 Apr 24;199(6):525–35. doi: 10.1007/s00066-023-02063-z (PMC10212877; doi:10.1007/s00066-023-02063-z)
Supplement: Supplementary file 2 — eTables 1–6 and eMethods. Supplemental tables include PICOS inclusion criteria, the risk of bias assessment for randomized design and non-randomized studies, details of the included studies, and results of meta regression analyses, respectively [file 66_2023_2063_MOESM2_ESM.docx]

**Supplemental Tables Content**

**[eTable 1.](#_Toc19673)** [PICOS](#_Toc19673)

**[eTable 2.](#_Toc14265)** [Risk of bias assessment for randomized design studies performed with Cochrane risk of bias tool (RoB2)](#_Toc14265)

**[eTable 3.](#_Toc7452)** [Risk of bias assessment for non-randomized studies performed with methodological index for non-randomized studies (MINORS)](#_Toc7452)

**[eTable 4.](#_Toc9439)** [Included studies of single-fraction high-dose-rate brachytherapy in localized prostate cancer](#_Toc9439)

**[eTable 5.](#_Toc10245)** [Details of grade 2 toxic effects and HDRB therapeutic schemes](#_Toc10245)

**[eTable 6.](#_Toc18612)** [Meta regression analysis](#_Toc18612)

**[eTable 6a.](#_Toc15346)** [Meta-regression analysis of variables related to bRFS at 3 year](#_Toc15346)

**[eTable 6b.](#_Toc10051)** [Meta-regression analysis of variables related to bRFS at 5 year](#_Toc10051)

**[eMethods:](#_Toc1888)** [R codes used to analyze each outcome measure](#_Toc1888)

**eTable 1. PICOS**

**Population,** **Intervention,** **Control,** **Outcome,** **Study** **Design** **(PICOS)** **Inclusion**

**Criteria.**

| **Population** | Patients with localized prostate cancer, who were primarily treated with HDRB radiotherapy. |
| --- | --- |
| **Intervention** | single dose of HDRB(defined as ≥ 15Gy/fraction) with or without ADT. |
| **Control** | Either no control group (such as single-arm study) or a multi-arm study which includes single dose group of HDRB and multiple doses group of HDRB (defined as > 1 fraction, < 15Gy/fraction) with or without ADT. |
| **Outcomes** | 3-year bRFS; 5-year bRFS; ≥ Grade 3 GU/GI toxicity; Grade 2 GU/GI toxicity. |
| **Study** **design** | Randomized trials, prospective studies, retrospective studies, studies with less than 15 patients will be excluded. |

Abbreviations: HDRB, High dose rate brchytherapy; Gy, gray; ADT, androgen deprivation therapy; bRFS, biochemical recurrence-free survival; GU, genitourinary; GI, gastrointestinal.

**eTable 2. Risk of bias assessment for randomized design studies performed with Cochrane risk of bias tool (RoB2)**

| **Study** | **Randomization process** | **Deviations from intended interventions** | **Mising outcome data** | **Measurement of the outcome** | **Selection of the reported result** | **Overall Bias** |
| --- | --- | --- | --- | --- | --- | --- |
| Corkum2021[27] | Low | Some concerns | Low | Low | Low | Some concerns |
| Morton2020[37] | Low | Some concerns | Low | Low | Low | Some concerns |
| Morton2017[36] | Low | Some concerns | Low | Low | Low | Some concerns |
| Hathout2019[10] | Low | Low | Low | Low | Low | Low |
| Reynaud2021[41] | Low | Low | Low | Low | Low | Low |
| Jolicoeur2021[33] | Some concerns | High | Low | Low | Some concerns | High |

**eTable 3. Risk of bias assessment for non-randomized studies performed with methodological index for non-randomized studies (MINORS)**

| **Methodological items for non-randomized studies** | **Score of Study*** | | | | | | |
| --- | --- | --- | --- | --- | --- | --- | --- |
|  | Armstrong2021[7]^#^ | Alayed2019[25] | Alayed2021[6]^#^ | Gomez-Iturriaga2020[28] | Gomez-Iturriaga2018[29] | Hoskin2017[31]^#^ | Hoskin2014[32]^#^ |
| 1. A clearly stated aim | 2 | 2 | 2 | 1 | 2 | 2 | 2 |
| 2. Inclusion of consecutive patients | 2 | 2 | 2 | 2 | 2 | 1 | 1 |
| 3. Prospective collection of data | 2 | 2 | 2 | 1 | 2 | 2 | 2 |
| 4. Endpoints appropriate to the aim of the study | 2 | 2 | 2 | 2 | 2 | 2 | 2 |
| 5. Unbiased assessment of the study endpoint | 1 | 2 | 1 | 1 | 1 | 2 | 1 |
| 6. Follow-up period appropriate to the aim of the study | 2 | 1 | 2 | 2 | 1 | 2 | 1 |
| 7. Loss to follow up less than 5% | 1 | 1 | 1 | 1 | 1 | 1 | 2 |
| 8. Prospective calculation of the study size | 0 | 0 | 0 | 0 | 0 | 0 | 0 |
| *Additional* *criteria* *in* *the* *case* *of* *comparative* *study* |  |  |  |  |  |  |  |
| 9. An adequate control group | 1 |  | 1 |  |  | 1 | 1 |
| 10. Contemporary groups | 2 |  | 0 |  |  | 2 | 2 |
| 11. Baseline equivalence of groups | 2 |  | 1 |  |  | 1 | 1 |
| 12. Adequate statistical analyses | 2 |  | 2 |  |  | 2 | 2 |
| Total | 19 | 12 | 16 | 10 | 11 | 18 | 17 |

*The items are scored 0 (not reported), 1 (reported but inadequate) or 2 (reported and adequate). The global ideal score being 16 for non-comparative studies and 24 for comparative studies. #The study had two or more groups.

**eTable 3. (continued) Risk of bias assessment for non-randomized studies performed with methodological index for non-randomized studies (MINORS)**

| **Methodological items for non-randomized studies** | **Score of Study*** | | | | | | | |
| --- | --- | --- | --- | --- | --- | --- | --- | --- |
|  | Siddiqui2019[42] | Krauss2017[35] | Krauss2021[34] | Prada2016[39] | Prada2018[40] | Soatti2021[43]^#^ | Xu2019[46] | Tharmalingam2020[44] |
| 1. A clearly stated aim | 2 | 2 | 2 | 2 | 2 | 2 | 2 | 2 |
| 2. Inclusion of consecutive patients | 2 | 2 | 2 | 1 | 1 | 1 | 1 | 2 |
| 3. Prospective collection of data | 2 | 2 | 1 | 2 | 2 | 2 | 2 | 2 |
| 4. Endpoints appropriate to the aim of the study | 2 | 2 | 2 | 2 | 2 | 2 | 2 | 2 |
| 5. Unbiased assessment of the study endpoint | 1 | 1 | 1 | 2 | 2 | 1 | 2 | 2 |
| 6. Follow-up period appropriate to the aim of the study | 2 | 1 | 2 | 2 | 2 | 1 | 1 | 1 |
| 7. Loss to follow up less than 5% | 1 | 1 | 1 | 2 | 2 | 1 | 1 | 1 |
| 8. Prospective calculation of the study size | 0 | 0 | 0 | 0 | 0 | 0 | 0 | 0 |
| *Additional* *criteria* *in* *the* *case* *of* *comparative* *study* |  |  |  |  |  |  |  |  |
| 9. An adequate control group |  |  |  |  |  | 1 |  |  |
| 10. Contemporary groups |  |  |  |  |  | 1 |  |  |
| 11. Baseline equivalence of groups |  |  |  |  |  | 1 |  |  |
| 12. Adequate statistical analyses |  |  |  |  |  | 2 |  |  |
| Total | 12 | 11 | 11 | 13 | 13 | 15 | 11 | 12 |

*The items are scored 0 (not reported), 1 (reported but inadequate) or 2 (reported and adequate). The global ideal score being 16 for non-comparative studies and 24 for comparative studies. #The study had two or more groups.

**eTable 3. (continued) Risk of bias assessment for non-randomized studies performed with methodological index for non-randomized studies (MINORS)**

| **Methodological items for non-randomized studies** | **Score of Study*** | | | |
| --- | --- | --- | --- | --- |
|  | Tsang2021[45]^#^ | Hannoun-Levi2022[30] | Barnes2019[26] | Peters2019[38] |
| 1. A clearly stated aim | 2 | 2 | 2 | 2 |
| 2. Inclusion of consecutive patients | 2 | 2 | 2 | 2 |
| 3. Prospective collection of data | 2 | 1 | 1 | 2 |
| 4. Endpoints appropriate to the aim of the study | 2 | 2 | 2 | 2 |
| 5. Unbiased assessment of the study endpoint | 2 | 2 | 2 | 2 |
| 6. Follow-up period appropriate to the aim of the study | 2 | 2 | 1 | 2 |
| 7. Loss to follow up less than 5% | 1 | 1 | 1 | 1 |
| 8. Prospective calculation of the study size | 0 | 0 | 0 | 0 |
| *Additional* *criteria* *in* *the* *case* *of* *comparative* *study* |  |  |  |  |
| 9. An adequate control group | 2 |  |  |  |
| 10. Contemporary groups | 2 |  |  |  |
| 11. Baseline equivalence of groups | 1 |  |  |  |
| 12. Adequate statistical analyses | 1 |  |  |  |
| Total | 19 | 12 | 11 | 13 |

*The items are scored 0 (not reported), 1 (reported but inadequate) or 2 (reported and adequate). The global ideal score being 16 for non-comparative studies and 24 for comparative studies. ^#^The study had two or more groups.

**eTable 4. Included studies of single-fraction high-dose-rate brachytherapy in localized prostate cancer**

| Author(s), year | Design | Patients | Dose | IPSS score | Prostate volume | Dose constraints | |
| --- | --- | --- | --- | --- | --- | --- | --- |
|  |  |  |  |  |  | Target region | Organs at risks |
| Armstrong2021[7] | PCS | Trial 1: 25  Trial 2: 25 | 21Gy(DIL)/1fx | Trial 1:  7 (2–18)  Trial 2:  5 (0–24) | Trial1:  48 (18–102.4) Trial2:  44.8(25.6–139.1) | Trial 1: PTVboost V100% > 95% nonboost V15Gy > 95% 65% < nonPTVboost V19Gy < 75%  Trial 2: boost V100% > 95% nonPTVboost V19Gy < 50% nonboost V15Gy > 95% | Rectum: V19Gy=0 D2.0cc (Gy) < 15 Gy  Urethra: D30(Gy) < 20.8 Gy D10 (Gy) < 22 Gy V150 (cm3) = 0 |
| Corkum2021[27] | RT | 87 | 19Gy/1fx | 4 (2–7) | < 60 | NR | NR |
| Morton2020[37] | RT | 87 | 19Gy/1fx | NR | < 60 | V100 > 95% V150 < 35% V200 < 12% | Rectum: Dmax < 90% V80 < 0.2 cc  Urethra: Dmax < 120% D10 < 115% |
| Morton2017[36] | RT | 87 | 19Gy/1fx | < 18 | 35 (27, 41) | V100 > 95% V150 < 35% V200 < 12% | Rectum: Dmax < 90% V80 < 0.2 cc  Urethra: Dmax < 120% D10 < 115% |
| Alayed2019[25] | PCS | 60 | 19Gy/1fx with DIL boost | 4 (0, 15) | 36 (17, 55) | DIL D90% ≥ 23 Gy  PTV: V100% >95% V150 < 35% V200 < 12% | Rectum: V80% <0.5 cc  Urethra: D10cc <118% |
| Alayed2021[6] | RCS | Trial 1: 87  Trial 2: 60 | 19Gy/1fx with or without DIL boost | Trial 1:  4 (2, 7) Trial 2:  3.5 (2, 8) | Trial 1:  35 (27, 40.3)  Trial 2:  34.9 (28, 42.5) | Trial 1: V100 > 95% V150 < 35% V200 < 12%  Trial2: DIL D90%≥23 Gy V100% > 95% V150% < 35%  V200% < 12% | Rectum: Trial 1: Dmax < 90% V80 < 0.2 cc Trial2: V80% <0.5 cc  Urethra: Trial 1: Dmax < 120% D10 < 115% Trial2: D10cc <118% |
| Gomez-Iturriaga2020[28] | PCS | 44 | 19Gy/1fx | 5 (0-14) | 34 (17-60) | V100 >95% V150 < 35% V200 < 8% | Rectum: D1cc < 60% Urethra: Dmax <110% |
| Gomez-Iturriaga2018[29] | PCS | 43 | 19Gy/1fx | 5 (0-14) | 34 (17-60) | V100 >95% 25% < V150 <35% V200 < 8% | Rectum: D1cc < 60% Urethra: Dmax <110% |
| Hathout2019[10] | RT | 16 | 19Gy/1fx | 5 (1-8) | 54.3 (35.5-143) | 105% < D90 < 115% V150 < 35% V200 < 12% | Rectum: V80 < 0.2 ml Dmax <90%  Urethra: D10 < 115% Dmax <120% |
| Reynaud2021[41] | RT | 16 | 19Gy/1fx | 5 (1–8) | 54.3 (35.5-143) | 105% < D90 < 115% V150 < 35% V200 < 12% | Rectum: V80 < 0.2 ml Dmax <90%  Urethra: D10 < 115% Dmax <120% |
| Hoskin2017[31] | PCS | 49 | 19Gy or 20Gy/1fx | <8: 45%  8–19: 47% | NR | NR | Rectum: D2cc < 15Gy V100 <100%  Urethra: D30% < 20.8Gy D10% < 22Gy V150 = 0 |
| Hoskin2014[32] | PCS | 49 | 19Gy or 20Gy/1fx | 0-7: 22  8-19: 24 | NR | NR | Rectum: D2cc < 15Gy 28.5 Gy(cc) = 0  Urethra: D30% < 20.8Gy D10% < 22Gy V30 Gy = 0 |

**eTable 4. (continued) Included studies of single-fraction high-dose-rate brachytherapy in localized prostate cancer**

| Author(s), year | Design | Patients | Dose | IPSS score | Prostate volume | Dose constraints | |
| --- | --- | --- | --- | --- | --- | --- | --- |
|  |  |  |  |  |  | Target region | Organs at risks |
| Jolicoeur2021[33] | RT | 99 | 19.5Gy/1fx | 6.3 (1.9-10.7) | < 60 | Criteria for CTV coverage were reached in 100% of the fraction | The dose constraints were achieved except for the bladder dose which was exceeded accounting for less than 1% deviation on dosimetric plans |
| Siddiqui2019[42] | PCS | 68 | 19Gy/1fx | ≤ 12 | ≤ 50 | V100 > 95% | Rectum: Dmax ≤ 72.5% Urethra: V110 < 10% |
| Krauss2017[35] | PCS | 58 | 19Gy/1fx | ≤ 12 | 63 (43-73) | V100 > 95% 50% < V125 < 60% | Rectum: Dmax ≤ 72.5% Urethra: V110 < 10% |
| Krauss2021[34] | PCS | 26 | 21Gy/1fx | <12 | ≤ 50 | V100 > 95% V150 > 25% V125 < 55% | Rectum: Dmax ≤ 72.5% Urethra: V110 < 10% |
| Prada2016[39] | PCS | 60 | 19Gy/1fx | NR | 38 (16–73) | The V90, V100, V150, V200, and D90 were recorded | Rectum: Dmax ≤ 75% Urethra: Dmax ≤ 110% |
| Prada2018[40] | PCS | 60 | 20.5Gy/1fx | NR | 38 (16–73) | The V90, V100, V150, V200, and D90 were recorded | Rectum: Dmax ≤ 75% Urethra: Dmax ≤ 110% |
| Soatti2021[43] | RCS | 87 | 19-20Gy/1fx | NR | NR | D90 > 95% | Rectum: D2cc < 75% Bladder: D2cc < 80%  Urethra: V1 < 115% V10 < 110% |
| Xu2019[46] | RCS | 124 | 19Gy/1fx | 7 (4-13) | 33 (26-47) | V19Gy > 90% | Rectum: V75% < 1 cc Bladder: V75% < 1 cc  Urethra: V125% < 1 cc |
| Tharmalingam2020[44] | PCS | 441 | 19Gy/1fx | NR | NR | V19Gy > 95% | Rectum: D2cc <15 Gy Dmax < 19 Gy  Urethra: D10 < 22 Gy D30 < 20.8 Gy |
| Tsang2021[45] | RCS | 78 | 19Gy/1fx | NR | NR | A minimum peripheral dose of 19 Gy was prescribed to the PTV | Rectum: D2cc < 15 Gy Dmax < 19 Gy  Urethra: D10 < 22 Gy D30 < 20.8 Gy |
| Hannoun-Levi2022[30] | PCS | 33 | 20Gy/1fx | 5 (0–14) | 40.8 (18.7-84) | D90 ≥ 105% V100 ≥ 95% V150 ≤ 35% V200 ≤ 15% | Rectum: V85 ≤ 1% Urethra: V110 < 1% |
| Barnes2019[26] | RCS | 28 | 19Gy or 21Gy/1fx | 12 (2-29) | 34.3 (16.5-122) | V100 > 90% | 19 Gy regimen: Rectum and bladder: V75 < 1 cc V150 = 0 cc Urethra:V125 < 1 cc V150 = 0 cc  21 Gy regimen: Rectum and bladder: V70 < 1 cc V135 = 0 cc Urethra:V115 < 1 cc V135 = 0 cc |
| Peters2019[38] | PCS | 30 | 19Gy/1fx | 5 (4-7) | 40 (32.3-41.7) | CTV D95 ≥ 19 Gy or D90 ≥ 17 Gy | Rectum and bladder: D1cc < 12 Gy  Urethra: D10 < 21 Gy |

D90: minimal dose delivered to 90% of target volume n; Vn : fractional volume of the organ that receives n% of the prescribed dose; Urethral Dmax: maximum point dose inside the urethral volume; Rectum Dmax: maximum point dose inside the rectum volume; D1cc and D2cc: dose delivered to 1 cc and 2 cc of the organ at risk. Abbreviations: Gy, gray; PTV, planning target volume; CTV, clinical target volume; DIL, dominant intraprostatic lesion; fx, fraction; RCS, Retrospective cohort study; PCS, Prospective cohort study; RT, Randomized trial.

**eTable 5. Details of grade 2 toxic effects and HDRB therapeutic schemes**

| Study | HDRB therapeutic schemes | Grade 2 toxic effect details |
| --- | --- | --- |
| Armstrong et al,2021[7] | 21 Gy HDRB in a single fraction, two de-escalation prescription schedules based on V19Gy^#^ for PTVnon-boost regions | Acute toxicity: the rate of acute grade 2 urinary toxicity was 16%, with 6 patients requiring short-term catheterisation for urinary retention. No acute grade 2 GI toxicity reported.  Late toxicity: 14% patients at 60months experienced grade 2 GU toxicity, and 2% patients occurred grade 2 GI toxicity. |
| Morton et al,2017[36] and Corkum et al,2021[27] | 19 Gy HDRB in a single fraction | Acute toxicity: One each case of constipation (1) and proctitis (1). Six patients developed acute urinary retention following discharge requiring catheterization.  Late toxicity: four patients experienced Grade 2 GI toxicity (2 proctitis, 1 constipation, 1 diarrhoea). Late grade 2 GU toxicity included urinary frequency (11), urinary retention (26), urinary obstruction (2), and urinary urgency(2). |
| Alayed et al,2019[25] and  Alayed et al,2021[6] | 19 Gy HDRB in a single fraction and additional focal boost to DIL at least 23 Gy | Acute toxicity: 13 patients experienced grade 2 GU toxicity.  Late toxicity: 17 patients experienced grade 2 GU toxicity and 3 patients occurred grade 2 GI toxicity. |
| Gomez-Iturriaga et al,2018[29] | 19 Gy HDRB in a single fraction | Acute grade 2 GU toxicity: dysuria (2), urgency (1) and nocturia (1). Four patients presented late GU grade-2 toxicity: dysuria (2) and nocturia (2). No grade 2 GI toxicity occurred. |
| Hathout et al,2019[10] and  Reynaud et al,2021[41] | 19 Gy HDRB in a single fraction | Grade 2 GU toxicity: 13.3% of increased frequency at 1 month and 7.7% at 3 months, 13.3% of urinary obstruction at 1 month, and 6.7% of urgency at 6 months. One patient experienced grade 2 GI toxicity at 36 months. |
| Hoskin et al,2014[32] and  Hoskin et al,2017[31] | 19 Gy or 20 Gy HDRB in a single fraction | No acute grade 2 GU toxicity and late grade 2 GI toxicity. One patient experienced acute grade 2 GI toxicity and 2.4% experienced late grade 2 GU toxicity. |
| Jolicoeur et al,2021[33] | 19.5 Gy HDRB in a single fraction | 7% in week1, 1% in week6, 2% at 3 months, 4% at 6 months, and 10% at 12 months experienced grade 2 GU toxicity. No grade 2 GI toxicity occurred. |
| Krauss et al,2017[35] and  Siddiqui et al,2019[42] | 19 Gy HDRB in a single fraction | Acute toxicity: frequency/urgency (4), followed by dysuria(2), hematuria (2), and retention (1). No acute grade 2 GI toxicity.  Late toxicity: urinary frequency/urgency (7), dysuria(1), hematuria (3), retention (1), incontinence(1), proctitis (1) and rectal bleeding (2). |
| Krauss et al,2021[34] | 21 Gy HDRB in a single fraction | Acute grade 2 urinary toxicity rates were 27% , with the corresponding chronic rates measuring 35%. |
| Prada et al,2016[39] | 19 Gy HDRB in a single fraction | Grade 2 toxicity was not observed in any patient. |
| Prada et al,2018[40] | 20.5 Gy HDRB in a single fraction | Grade 2 toxicity was not observed in any patient. |
| Xu et al,2019[46] | 19-20 Gy HDRB in a single fraction | Of 99 patients with GU late toxicity grades, 59 had Grade 2 toxicities. |
| Tharmalingam et al,2020[44] | 19 Gy HDRB in a single fraction | Acute grade 2 GU and GI toxicity peaked at 1 month post-implant; rates of 12% and 3% respectively. Acute urinary retention requiring catherization occurred in 16 patients. |
| Tsang et al,2021[45] | 19 Gy HDRB in a single fraction | Grade 2 GU toxicities were observed in 20 patients and no Grade 2 GI toxicities occurred. |
| Hannoun-Levi et al,2022[30] | 20 Gy HDRB in a single fraction | Acute GU and GI toxicities were observed in 8 and 3 patients. No grade ≥ 2 late toxicity was observed. |
| Barnes et al,2019[26] | 19 Gy or 21 Gy HDRB in a single fraction | Acute toxicity: urinary frequency (5), urinary urgency (3), dysuria (1), and retention (2). Five patients reported late toxicities, involving urinary frequency (5), urinary urgency (3), dysuria (1), and retention (1). No patients experienced GI toxicities. |
| Peters et al,2019[38] | 19 Gy HDRB in a single fraction | Intraoperative haemorrhage occurred in 1 patient, caused by premature retraction of an unfolded umbrella catheter. This patient developed an acute urinary retention (grade 2). Grade 2 GU toxicity developed in other 4 patients, including urinary frequency (3) and cystitis (1). No grade 2 GI toxicity occurred. |

# Percentage of prostate volume receiving a dose of 19 Gy. Abbreviations: Gy, gray; HDRB, high-dose-rate brachytherapy; GU, genitourinary; GI, gastrointestinal; PTV, planning target volume; DIL, dominant intraprostatic lesion; MRI, magnetic resonance imaging.

**eTable 6. Meta regression analysis**

**eTable 6a. Meta-regression analysis of variables related to bRFS at 3 year**

| Variables | Studies | Coefficients | Lower bound | Upper bound | P value |
| --- | --- | --- | --- | --- | --- |
| Intercept | 14 | 1.0660 | 0.9354 | 1.1967 | <.0001 |
| Proportion of ADT receipt |  | 0.1733 | -0.0533 | 0.4000 | 0.1192 |
| Proportion of gleason score ≥7 |  | 0.2136 | -0.0030 | 0.4301 | 0.0527 |
| Proportion of clinical T stage ≥T2b |  | -0.1150 | -0.3792 | 0.1493 | 0.3553 |

I^2^ (residual heterogeneity / unaccounted variability): 24.3%

R^2^ (amount of heterogeneity accounted for): 53.30%

Test for Residual Heterogeneity: QE (df = 10) = 11.8844, p-val = 0.2929

Test of Moderators (coefficients 2:4): F (df1 = 3, df2 = 10) = 3.0885, p-val = 0.0767

**eTable 6b. Meta-regression analysis of variables related to bRFS at 5 year**

| Variables | Studies | Coefficients | Lower bound | Upper bound | P value |
| --- | --- | --- | --- | --- | --- |
| Intercept | 7 | 0.9923 | 0.7334 | 1.2512 | 0.0012 |
| Proportion of ADT receipt |  | 0.1523 | -0.2516 | 0.5561 | 0.3163 |
| Proportion of gleason score ≥7 |  | -0.0279 | -0.6155 | 0.5597 | 0.8895 |
| Proportion of clinical T stage ≥T2b |  | 0.0445 | -0.8023 | 0.8914 | 0.8778 |

I^2^ (residual heterogeneity / unaccounted variability): 40.98%

R^2^ (amount of heterogeneity accounted for): 0.00%

Test for Residual Heterogeneity: QE (df = 3) = 4.9936, p-val = 0.1723

Test of Moderators (coefficients 2:4): F(df1 = 3, df2 = 3) = 0.9602, p-val = 0.5129

**eMethods: R codes used to analyze each outcome measure**

**##Code to generate Cumulative Occurrence of Grade 3-5 GI Toxicity Forest and Funnel Plots**

*```{r install packages} install.packages(c("meta","metafor")) ```*

*```{r load packages} library(meta) library(metafor)*

*```{r calculate overall summary proportion using meta package}*

view(Cumulative_occurrence_of_grade_3_GI_toxicity)

dat<- Cumulative_occurrence_of_grade_3_GI_toxicity

m<- metaprop(cases,patients,study,data=dat,sm="PAS",method.tau="REML",method.ci

="NAsm",incr=0.5,allincr=FALSE,addincr=FALSE,hakn=TRUE,control=list(stepadj=0.5, maxiter=100))

summary(m)

*```{r generate forest plot using meta package}*

forest(m, xlim = c(0,16), pscale =100, fixed=FALSE,random = TRUE, lty.fixed=2, lty.random=2, type.study="square",type.random="diamond",ff.fixed="bold.italic", ff.random="bold",ff.predict = "bold",ff.xlab = "bold",hetlab = "Heterogeneity:",seTE.predict=TRUE,rightcols= FALSE,leftcols = c("studlab", "patients","Median dose","effect","ci"),leftlabs = c("Source","patients","Median Dose","Events rate,%","[95%CI]"), xlab = "Grade 3-5 GI toxic effects(%)",just.addcols.left = "left",squaresize=0.5,col.square="black",col.square.lines="black",col.diamond="black",col.diamond.lines="black",print.Q=TRUE,print.pval.Q=TRUE,print.I2=TRUE,print.I2.ci=TRUE,print.tau2=TRUE,print.tau2.ci=TRUE,calcwidth.fixed=TRUE,print.tau=TRUE,print.tau.ci=TRUE,calcwidth.pooled=TRUE,calcwidth.hetstat=FALSE,weight.study="random",col.by="grey",digits=1,digits.tau2=2,digits.tau=2,colgap.forest.left="0.5inch",colgap.forest.right="0inch",addrow=0,ref=0,fs.random=12,fs.predict=12,fs.hetstat=12,fs.axis=12,fs.xlab=12,fs.study=12,fs.study.lables=12,prediction=TRUE,fs.heading=12,smlab = "",col.inside = "black")

*```{r conduct sensitivity analysis using meta}*

metainf(m, pooled = "random")

forest(metainf(m, pooled = "random"),digits = 4,random = TRUE,xlab = "Grade 3-5 GI toxic effects")

*```{r perform subgroup analysis using meta}*

sub=update(m,subgroup=median_dose)

sub

forest (sub)

*```{r generate funnel plot using metafor}*

m1=escalc(xi=cases,ni=patients,measure="PAS",data=dat)

res=rma(yi,vi,data=m1,method="REML",test="knha",control=list(stepadj=0.5, maxiter=100))

funnel(res,atransf=transf.iarcsin,yaxis="sei",xlab="Proportion",digits=4,level=c(90,95,99),shade=c("white", "gray55", "gray75"), legend=TRUE)

*```{r conduct Egger Linear Regression Test using metafor}*

regtest(res,model = "rma",digits = 2)

**##Code to generate Cumulative Occurrence of Grade 3-5 GU Toxicity Forest and Funnel Plots**

*```{r install packages} install.packages(c("meta","metafor")) ```*

*```{r load packages} library(meta) library(metafor)*

*```{r calculate overall summary proportion using meta package}*

view(Cumulative_occurrence_of_grade_3_GU_toxicity)

dat<-Cumulative_occurrence_of_grade_3_GU_toxicity

m<-metaprop(cases,patients,study,data=dat,sm="PAS",method.tau="REML",method.ci ="NAsm",incr=0.5,allincr=FALSE,addincr=FALSE,hakn=TRUE)

summary(m)

*```{r generate forest plot using meta package}*

forest(m, xlim = c(0,16), pscale =100, fixed=FALSE,random = TRUE, lty.fixed=2, lty.random=2, type.study="square",type.random="diamond",ff.fixed="bold.italic", ff.random="bold",ff.predict = "bold",ff.xlab = "bold",hetlab = "Heterogeneity:",seTE.predict=TRUE,rightcols= FALSE,leftcols = c("studlab", "patients","Median dose","effect","ci"),leftlabs = c("Source","patients","Median Dose","Events rate,%","[95%CI]"), xlab = "Grade 3-5 GU toxic effects(%)",just.addcols.left = "left",squaresize=0.5,col.square="black",col.square.lines="black",col.diamond="black",col.diamond.lines="black",print.Q=TRUE,print.pval.Q=TRUE,print.I2=TRUE,print.I2.ci=TRUE,print.tau2=TRUE,print.tau2.ci=TRUE,calcwidth.fixed=TRUE,print.tau=TRUE,print.tau.ci=TRUE,calcwidth.pooled=TRUE,calcwidth.hetstat=FALSE,weight.study="random",col.by="grey",digits=1,digits.tau2=2,digits.tau=2,colgap.forest.left="0.5inch",colgap.forest.right="0inch",addrow=0,ref=0,fs.random=12,fs.predict=12,fs.hetstat=12,fs.axis=12,fs.xlab=12,fs.study=12,fs.study.lables=12,prediction=TRUE,fs.heading=12,smlab = "",col.inside = "black")

*```{r conduct sensitivity analysis using meta}*

metainf(m, pooled = "random")

forest(metainf(m, pooled = "random"),digits = 4,random = TRUE,xlab = "Grade 3-5 GU toxic effects")

*```{r perform subgroup analysis using meta}*

sub=update(m,subgroup=median_dose)

sub

forest (sub)

*```{r generate funnel plot using metafor}*

m1=escalc(xi=cases,ni=patients,measure="PAS",data=dat)

res=rma(yi,vi,data=m1,method="REML",test="knha")

funnel(res,atransf=transf.iarcsin,yaxis="sei",xlab="Proportion",digits=4,level=c(90,95,99),shade=c("white", "gray55", "gray75"), legend=TRUE)

*```{r conduct Egger Linear Regression Test using metafor}*

regtest(res,model = "rma",digits = 2)

**##Code to generate 3-year bRFS Forest and Funnel Plots**

*```{r install packages} install.packages(c("meta","metafor")) ```*

*```{r load packages} library(meta) library(metafor)*

*```{r calculate overall summary proportion using meta package}*

view(X3_year_bRFS_data)

dat<-X3_year_bRFS_data

m<-metaprop(cases,patients,study,data=dat,sm="PAS",method.tau="REML",method.ci="NAsm",incr=0.5,allincr=FALSE,addincr=FALSE,hakn=TRUE)

summary(m)

*```{r generate forest plot using meta package}*

forest(m, xlim = c(20,100), pscale =100, fixed=FALSE,random = TRUE, lty.fixed=2,lty.random=2, type.study="square",type.random="diamond",ff.fixed="bold.italic", ff.random="bold",ff.predict = "bold",ff.xlab = "bold",hetlab = "Heterogeneity:",seTE.predict=TRUE,rightcols= FALSE,leftcols = c("studlab", "patients","Median dose","effect","ci"),leftlabs = c("Source","patients","Median Dose","3-year bRFS,%","[95%CI]"), xlab = "3-year bRFS(%)",just.addcols.left = "left",squaresize =0.5,col.square="black",col.square.lines="black",col.diamond="black",col.diamond.lines="black",print.Q=TRUE,print.pval.Q=TRUE,print.I2=TRUE,print.I2.ci=TRUE,print.tau2=TRUE,print.tau2.ci=TRUE,calcwidth.fixed=TRUE,print.tau=TRUE,print.tau.ci=TRUE,calcwidth.pooled=TRUE,calcwidth.hetstat=FALSE,weight.study="random",col.by="grey",digits=1,digits.tau2=2,digits.tau=2,digits.pval.Q=2,colgap.forest.left="0.5inch",colgap.forest.right="0inch",addrow=0,ref=20,fs.random=12,fs.predict=12,fs.hetstat=12,fs.axis=12,fs.xlab=12,fs.study=12,fs.study.lables=12,prediction=TRUE,fs.heading=12,smlab = "",spacing=1.1)

*```{r conduct sensitivity analysis using meta}*

metainf(m, pooled = "random")

forest(metainf(m,pooled="random"))

*```{r perform subgroup analysis using meta}*

sub=update(m,subgroup=median_dose)

sub

forest (sub)

sub=update(m,subgroup=Risk_group)

sub

forest (sub)

*```{r perform meta regression using metafor}*

m1=escalc(xi=cases,ni=patients,measure="PAS",data=dat)

res=rma(yi,vi,data=m1,method="REML",test="knha")

reg1=rma(yi,vi,data=m1,method="REML",mods = ~ dat$`Proportion of ADT receipt`+dat$`Proportion of gleason score ≥7`+dat$`Proportion of clinical T stage ≥T2b`,test="knha")

reg1

*```{r generate funnel plot and using metafor}*

funnel(res,atransf=transf.iarcsin,yaxis="sei",xlab="Proportion",digits=4,level=c(90,95,99),shade=c("white", "gray55", "gray75"), legend=TRUE)

*```{r conduct Egger Linear Regression Test using metafor}*

regtest(res,model = "rma",digits = 2)

**##Code to generate 5-year bRFS Forest and Funnel Plots**

*```{r install packages} install.packages(c("meta","metafor")) ```*

*```{r load packages} library(meta) library(metafor)*

*```{r calculate overall summary proportion using meta package}*

view(X5_year_bRFS_data)

dat<-X5_year_bRFS_data

m<-metaprop(cases,patients,study,data=dat,sm="PAS",method.tau="REML",method.ci="NAsm",incr=0.5,allincr=FALSE,addincr=FALSE,hakn=TRUE)

summary(m)

*```{r generate forest plot using meta package}*

forest(m, xlim = c(20,100), pscale =100, fixed=FALSE,random =TRUE,lty.fixed=2,lty.random=2, type.study="square",type.random="diamond",ff.fixed="bold.italic", ff.random="bold",ff.predict = "bold",ff.xlab = "bold",hetlab = "Heterogeneity:",seTE.predict=TRUE,rightcols= FALSE,leftcols = c("studlab", "patients","Median dose","effect","ci"),leftlabs = c("Source","patients","Median Dose","5-year bRFS,%","[95%CI]"), xlab = "5-year bRFS(%)",just.addcols.left = "left",squaresize =0.5,col.square="black",col.square.lines="black",col.diamond="black",col.diamond.lines="black",print.Q=TRUE,print.pval.Q=TRUE,print.I2=TRUE,print.I2.ci=TRUE,print.tau2=TRUE,print.tau2.ci=TRUE,calcwidth.fixed=TRUE,print.tau=TRUE,print.tau.ci=TRUE,calcwidth.pooled=TRUE,calcwidth.hetstat=FALSE,weight.study="random",col.by="grey",digits=1,digits.tau2=2,digits.tau=2,digits.pval.Q=2,colgap.forest.left="0.5inch",colgap.forest.right="0inch",addrow=0,ref=20,fs.random=12,fs.predict=12,fs.hetstat=12,fs.axis=12,fs.xlab=12,fs.study=12,fs.study.lables=12,prediction=TRUE,fs.heading=12,smlab = "",spacing = 1.1)

*```{r conduct sensitivity analysis using meta}*

metainf(m, pooled = "random")

forest(metainf(m,pooled="random"))

*```{r perform subgroup analysis using meta}*

sub=update(m,subgroup=median_dose)

sub

forest(sub)

sub=update(m,subgroup=Risk_group)

sub

forest(sub)

*```{r perform meta regression using metafor}*

m1=escalc(xi=cases,ni=patients,measure="PAS",data=dat)

res=rma(yi,vi,data=m1,method="REML",test="knha")

reg1=rma(yi,vi,data=m1,method="REML",mods = ~ dat$`Proportion of ADT receipt`+dat$`Proportion of gleason score ≥7`+dat$`Proportion of clinical T stage ≥T2b`,test="knha")

reg1

*```{r generate funnel plot using metafor}*

funnel(res,atransf=transf.iarcsin,yaxis="sei",xlab="Proportion",digits=4,level=c(90,95,99),shade=c("white", "gray55", "gray75"), legend=TRUE)

*```{r conduct Egger Linear Regression Test using metafor}*

regtest(res,model = "rma",digits = 2)
